# Improving LLM-based event extraction with annotation guidelines

**DOI:** 10.3389/frai.2026.1797435

**Published:** 2026-08-13

**Authors:** Marcel Geromel, Philipp Cimiano

**Affiliations:** Center for Cognitive Interaction Technology, Bielefeld University, Bielefeld, Germany

**Keywords:** event extraction, information extraction, LLM, prompt engineering, synthetic annotation

## Abstract

Event extraction constitutes a foundational task in information extraction, but reliance on laborious and expensive human annotations severely restricts the availability of training datasets. While recent works have explored Large Language Models (LLMs) as example-driven (or zero-shot) annotators, they are substantially outperformed by supervised techniques on structured extraction tasks, such as event detection and argument extraction, possibly on account of underspecified task instructions. In this work, we investigate to which extent LLMs can benefit from dataset-specific, detailed annotation guidelines that more precisely represent the dataset's underlying (human) annotation procedures. To this end, we propose a guideline-based, three-stage LLM annotation framework for event extraction that incorporates detailed event annotation guidelines and supports multiple LLM annotators to improve robustness. Using the comprehensive and well-documented ACE 2005 English Annotation Guidelines for Events as a reference document, we evaluate four LLMs across three guideline-compliant benchmark datasets. Our findings indicate that, depending on model choice, guideline specificity, and the dataset's relative label accuracy, employing detailed guidelines can considerably boost event extraction performance, gaining up to 6.7 F1 points over commonly used bare-minimum instructions, with particularly remarkable improvements for reasoning-based models. Furthermore, we demonstrate that augmenting existing datasets with LLM-generated argument annotations can improve argument extraction performance under soft-matching evaluation. Overall, our experiments emphasize the importance of annotation guidelines (as well as their specificity) for LLM-based annotations, providing valuable insights on leveraging LLMs as guideline-compliant annotators.

## 1 Introduction

Event extraction is an important information extraction task in natural language processing that aims to generate or retrieve structured information about specific events (e.g., *who did what to whom, where*, and *when*) from unstructured textual sources, laying the foundation for numerous downstream applications such as knowledge graph construction, summarization, and question answering (Guan et al., 2023).

Most previous research has approached event extraction and its subtasks—such as event detection and event argument extraction—primarily from a supervised learning perspective, including current state-of-the-art methods (Lu et al., 2022; Liu et al., 2023; Lou et al., 2023), requiring carefully annotated datasets for training. By training on labeled samples, supervised approaches acquire an implicit understanding of the annotation guidelines that governed the dataset's creation and labeling process. However, producing such ground-truth annotations can be laborious and expensive, especially for event extraction, where annotation guidelines are notoriously complex. Specifically, these guidelines often encompass a multitude of annotation details and criteria (Aguilar et al., 2014), may require multi-step reasoning, or demand extensive domain expertise (Kim et al., 2008), thus further limiting the amount and volume of high-quality annotated datasets, rendering event extraction even more challenging.

In recent years, this shortage of available training data has stimulated the development of various low-resource supervised learning approaches designed to generalize event extraction from a limited number of training samples (Hsu et al., 2022; Zhao et al., 2023; Duan et al., 2024), achieving remarkable performance in low-resource scenarios. To no surprise, these works also consistently demonstrate that the performance of (supervised) low-resource approaches can typically be improved by increasing the amount of training data available, especially for long-tailed events (Kan et al., 2024), showing that having more annotated data is generally desirable.

To acquire more training data, various recent studies have adopted prompt-based (both few- and zero-shot) methods to generate “synthetic” annotations (even complete datasets) using Large Language Models (LLMs) such as GPT-4 (OpenAI, 2024) as backbone annotators (Tan et al., 2024), showing huge success for relatively basic annotation tasks, such as stance and theme detection (Gilardi et al., 2023), question answering (Fischer et al., 2024), and named entity recognition (Naraki et al., 2024). However, when approaching more advanced extraction tasks (e.g., relation and event extraction), such prompt-driven methods are vastly outperformed by supervised techniques (Wei et al., 2024; Han et al., 2024), suggesting a performance inappropriate for labeling datasets (i.e., annotation).

An obvious limitation and potential explanation for the limited success of prompt-based event extraction methods may stem from the underspecified problem descriptions that are commonly presented as instructions to an LLM during inference. While these “annotation guidelines” typically describe all relevant aspects of *what* features to annotate, they seldom explain *how* (e.g., which rules to follow), leaving out important elements that would otherwise be conveyed to human annotators. Inspired by these findings, we intend to answer the following question: *To which degree can LLMs benefit from detailed event annotation guidelines?*

Therefore, we investigate the impact of prompting LLMs with exhaustive, real-world annotation guidelines on performance. We contribute the following:

(a) We propose a guideline-based, three-stage LLM prompting framework for event extraction. To ensure consistent and robust annotations, we develop various methods to aggregate different LLM predictions.
(b) We evaluate our approach by conducting experiments on three common event extraction datasets and four instruction-following LLMs, showcasing that detailed annotation guidelines can markedly improve model performance, boosting results by up to 6.7 points (F-score) over commonly used “bare-minimum” instructions.
(c) We use our LLM-generated annotations for training various supervised event extraction baselines, demonstrating that (1) training on such annotations when no labeled samples are available provides a strong baseline, and (2) enriching existing datasets with LLM-generated argument annotations can markedly improve results when evaluated with soft-matching.

## 2 Methods

### 2.1 Task description

We assume and follow the standard event extraction task formulation (Ahn, 2006):

Let *x* represent a token sequence *x*_1_, ..., *x*_*n*_, $N_{x}$ comprise any *non-empty* token spans {(*s, e*) ∈ ℕ^2^ ∣ 1 ≤ *s* < *e* ≤ |*x*|}, E denote a finite set of event types (e.g., *Conflict* and *Justice*), $S_{e}$ constitute the set of subtypes for each event type e∈E (e.g., *Conflict.Attack* and *Justice.Pardon*), and $R_{s}$ represent the set of argument roles for each event subtype $s\in S_{e}$ (e.g., *Attacker* and *Target*). Note: The elements $s\in S_{e}$ can be assumed to uniquely represent their respective event types e∈E, for example, through *Type.Subtype* notation. Therefore, we eliminate redundant mentions of event types whenever applicable.

In event extraction, two common subtasks are entailed:

- **Event Detection**—For an input sequence *x*, we seek to determine the respective event mentions
$$M\left(x\right)\subset \left\{\left(t,s\right)|t\in N_{x},s\in S_{e},e\in E\right\}$$
  where *t* denotes the event's *trigger* which is uniquely assigned to an event subtype:
$$\forall \left(t_{i},s_{i}\right),\left(t_{j},s_{j}\right)\in M\left(x\right):t_{i}=t_{j}\Rightarrow s_{i}=s_{j}$$
- **Argument Extraction**—For an event mention $\left(t_{i},s_{i}\right)\in M\left(x\right)$, we determine the respective arguments:
$$A_{i}\left(x\right)={\left\{\left(t_{i},s_{i},a_{i,j},r_{i,j}\right)\right\}}_{j=1}^{k_{i}}\;A\left(x\right)=\underset{m_{i}}{⋃}A_{i}\left(x\right)$$
  where $a_{i,j}\in N_{x}$ and $r_{i,j}\in R_{s_{i}}$ represent the argument's span and role.

**Figure 1:**
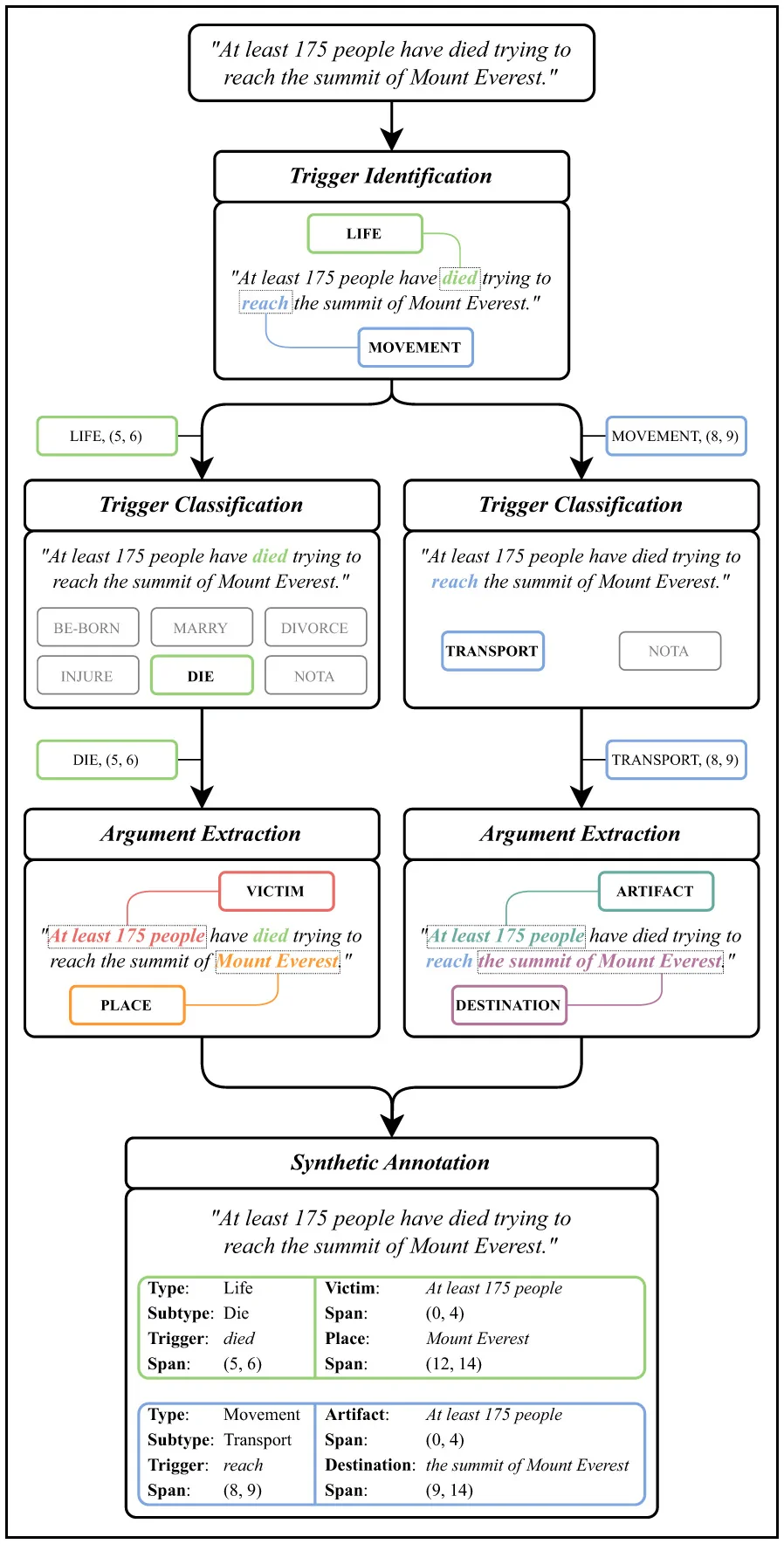
Our pipeline approach, illustrated. The example sentence contains two event mentions, *LIFE.DIE* and *MOVEMENT.TRANSPORT*.

We handle event detection in two phases: trigger identification and classification. Accordingly, our pipeline consists of three steps for event extraction: trigger identification, trigger classification, and argument extraction. The complete pipeline is illustrated in Figure 1.

### 2.2 Step 1: Trigger identification

In this step, given an input sequence *x*, we seek to generate a selection of candidate triggers $t_{i}\in N_{x}$ with corresponding event types $e_{i}\in E$. The candidate spans and event types (*t*_*i*_, *e*_*i*_) are predicted by prompting the annotator *A* (an LLM) to annotate the sequence *x*_1_, ..., *x*_*n*_ with opening and closing markers, such as <LIFE> and </LIFE>, to highlight potential event triggers.

**Figure 2:**
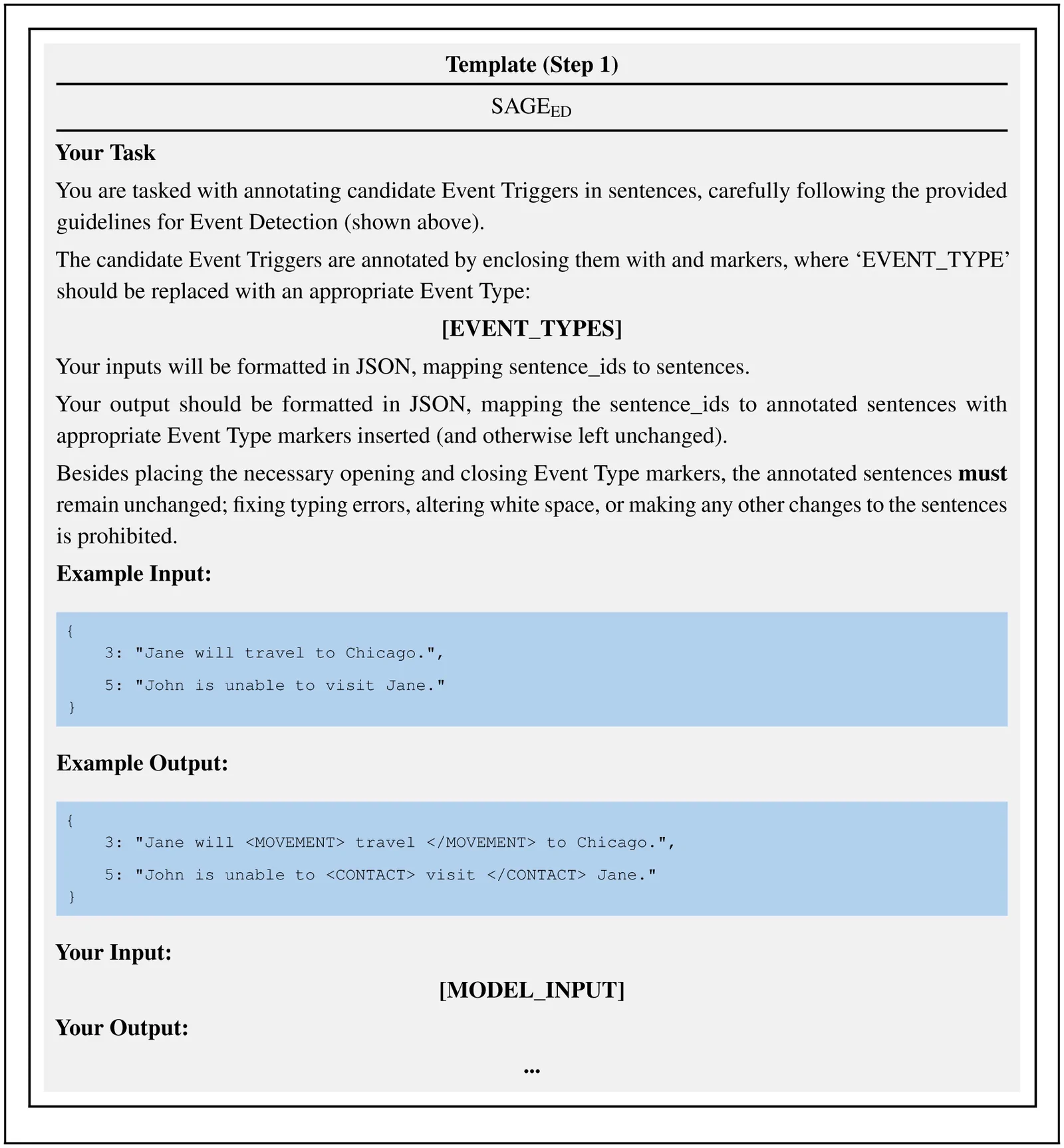
The instruction template for Step 1. The blue area contains a labeled example (fixed) for the model to demonstrate the desired output format. The [EVENT_TYPES] and [MODEL_INPUT] symbols are replaced at runtime.

To achieve this, the model is provided with (1) a guideline document with detailed instructions on annotating event mentions, (2) an explanation of the annotation task, with instructions on proper output formatting, and (3) the input sequence *x*. Our prompt is provided verbatim in Figure 2.

**Figure 3:**
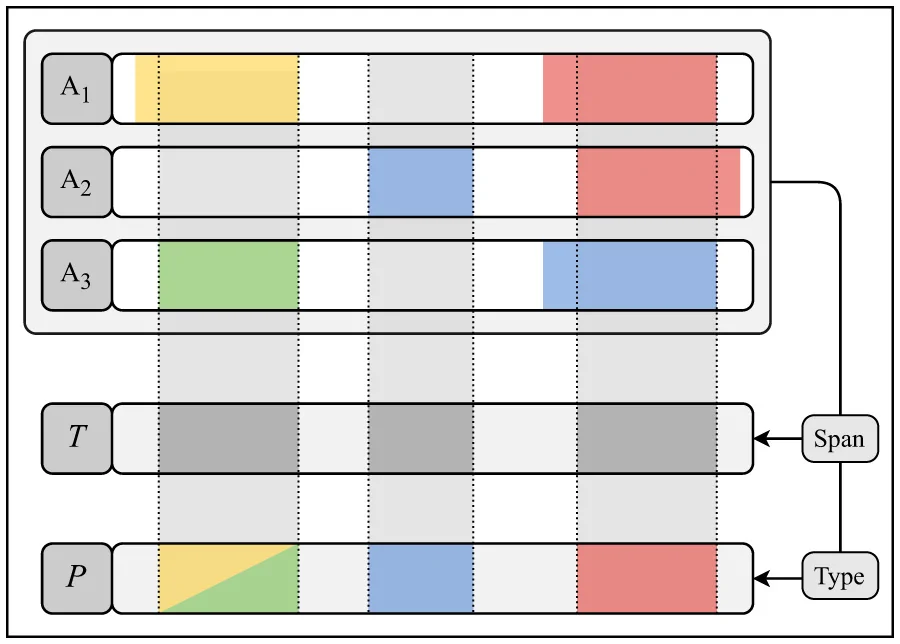
An illustration of the merging process in Step 1. The resulting candidate event spans T are determined from the preliminary predictions of *A*_1_, ..., *A*_3_ before the corresponding event types (P) are determined by finding the respective, most frequently predicted event type(s).

To obtain robust predictions, we simulate multiple annotations *A*_1_, ..., *A*_*k*_ by sampling *k* predictions from *A*, and aggregate the responses via the following procedure (see Figure 3):

Let *T* = *t*_1_, ..., *t*_*m*_ and *E* = *e*_1_, ..., *e*_*m*_ represent the preliminary event spans $t_{i}\in N_{x}$ with respective event types $e_{i}\in E$ obtained by sampling *A*_1_ through *A*_*k*_. For each position *p* ∈{1, ..., *n*} in sequence *x*_1_, ..., *x*_*n*_, we intersect the preliminary triggers *t*_*i*_ from (*t*_1_, ..., *t*_*m*_) that intersect with position *p*, which yields a set of maximal, disjoint candidate triggers T. For each span t∈T, we determine candidate event types ε(e)⊆E by voting.

Formally, we aggregate the candidate event spans through T={τ(p)∣p∈{1,...,n},σ(p)≠∅}, where σ(*p*) := {*t*_*i*_ ∈ (*t*_1_, ..., *t*_*m*_) ∣ *t*_*i*_ intersects *p*} denotes all preliminary event spans overlapping *p*, and τ(*p*) := (max{*a* ∣ (*a, b*) ∈ σ(*p*)}, min{*b*∣(*a, b*) ∈ σ (*p*)}) determines the intervals obtained by intersecting the event spans σ(*p*). The corresponding event types are established via ε(*t*) := ${\mathrm{argmax}}_{e\in E}|\left\{i\in \left\{1,...,m\right\}|t_{i}\text{intersects}t,e_{i}=e\right\}|$.

The final predictions for *Trigger Identification* are obtained thus:

$$P_{1}=\left\{\left(t,e\right)|t\in T,e\in \varepsilon \left(t\right)\right\}$$

### 2.3 Step 2: Trigger classification

Using the aggregated predictions $\left(t,e\right)\in P_{1}$ from Step 1, we determine the underlying event subtypes $s\in S_{e}$ for each candidate span *t* and corresponding type e∈E, where $S_{e}$ denotes the potential subtypes of event type *e*, for example, $S_{\text{Life}}=\left\{\text{Marry},\text{Injure},...\right\}$.

Specifically, for each tuple (*t, e*) in $P_{1}$, we formulate the decision problem (i.e., which event subtype *s* is represented) as a multiple choice question. To that end, we provide the annotator *A* (the LLM) with **(1)** a guideline document with detailed instructions on annotating event types and subtypes, **(2)** an explanation and instructions for answering the multiple choice question (e.g, output format), **(3)** the sequence *x*_1_, ..., *x*_*n*_, wherein the relevant trigger tokens (in *t*) are emphasized with event type markup symbols (as discussed in Step 1), for example, <LIFE> and </LIFE>, and **(4)** the available event subtypes $S_{e}$.

**Figure 4:**
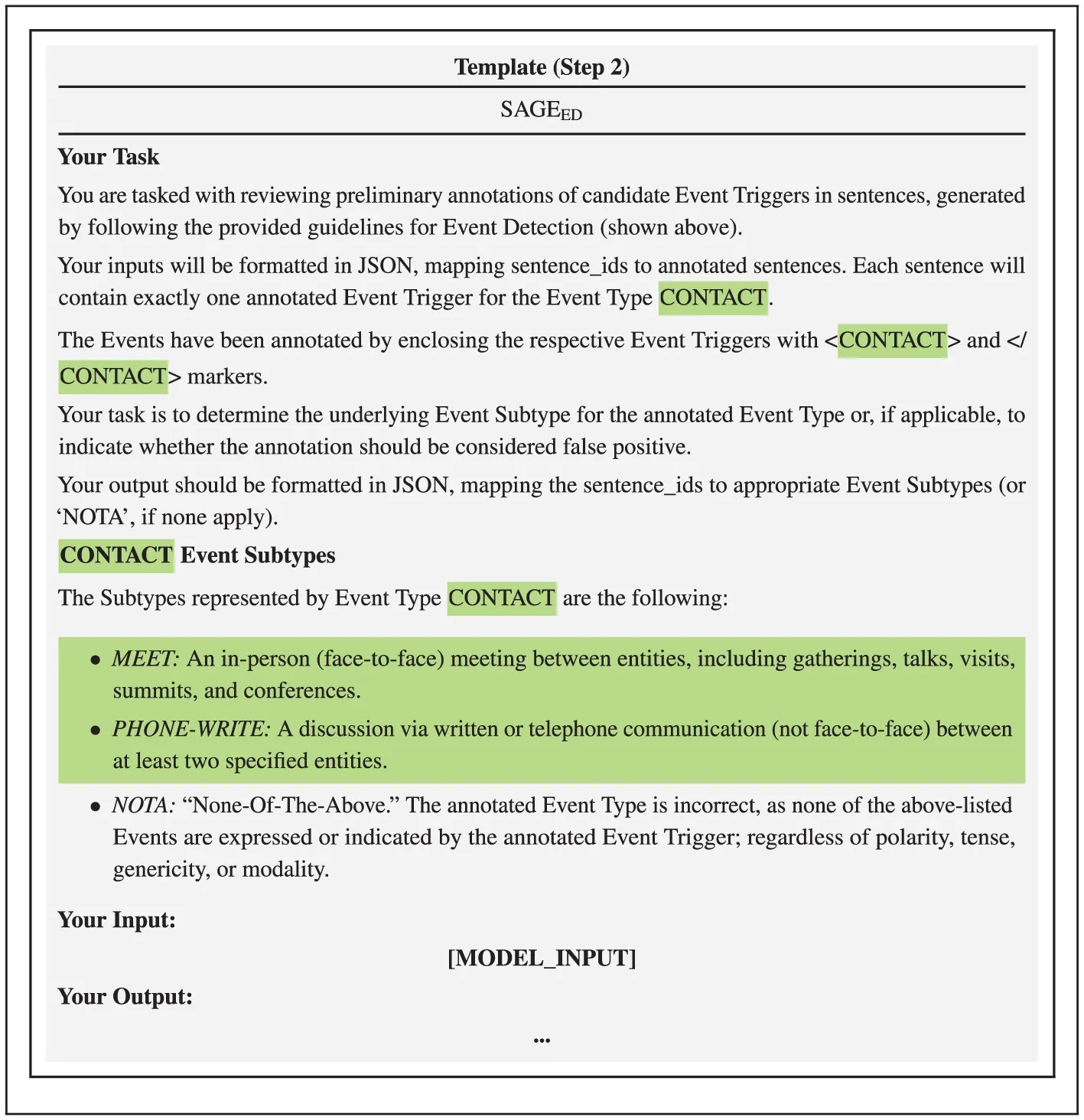
The instruction template for Step 2. The marked regions (green) are event type specific and comprise relevant context and definitions from §5 Event Types. The [MODEL_INPUT] placeholder is replaced at runtime.

From that, annotator *A* should choose a suitable event subtype *s* from the potential answers $S_{e}$ by following the annotation guidelines, or respond with *NOTA* (“none of the above”) if the emphasized trigger tokens *t* and event type *e* are assumed incorrect. Our prompt is presented in Figure 4.

Again, we simulate multiple annotations *A*_1_, ..., *A*_*k*_ by sampling *k* outputs from *A* to obtain robust predictions. The predictions are aggregated as follows:

Let $\pi \left(t,e\right)={ŝ}_{t,e}^{\left(1\right)},...,{ŝ}_{t,e}^{\left(k\right)}\in S_{e}\cup \left\{\text{NOTA}\right\}$ represent the predicted subtypes (or ‘NOTA') obtained by sampling *A*_1_ through *A*_*k*_ for (*t, e*) in $P_{1}$. We establish the subtype *s*_*i*_ by majority voting across π(*t, e*), where *s*_*i*_ = NOTA when no majority is obtained. Next, we eliminate NOTA predictions, obtaining *M* := $\left\{\left(t,s\right)|\left(t,e\right)\in P_{1},s\neq \text{NOTA}\right\}$.

Lastly, by removing duplicate event spans in *M*, we obtain our final predictions for *Trigger Classification*:

$P_{2}$ := {(*t*_*i*_, *s*_*i*_)∈*M*∣∀(*t*_*j*_, *s*_*j*_)∈*M*:*t*_*i*_ = *t*_*j*_⇒*s*_*i*_ = *s*_*j*_}

### 2.4 Step 3: Argument extraction

For each event span and subtype $\left(t,s\right)\in P_{2}$, we determine argument mentions $a\in N_{x}$ for the subtype-specific argument roles $R_{s}$, such as $R_{\text{Attack}}=\left\{\text{Agent},\text{Target},...\right\}$. Note that annotator *A* may generate more than one argument for a specific role $r\in R_{s}$. For instance, *Jane* married *John* (having *s* = *Marry*) implies two distinct *Person* arguments.

The argument spans with respective roles (*a*_*i*_, *r*_*i*_) are obtained by prompting *A* to enclose all required arguments by inserting markup symbols into sequence *x*, for example, <Person> and </Person>, and leaving the sequence otherwise unchanged.

**Figure 5:**
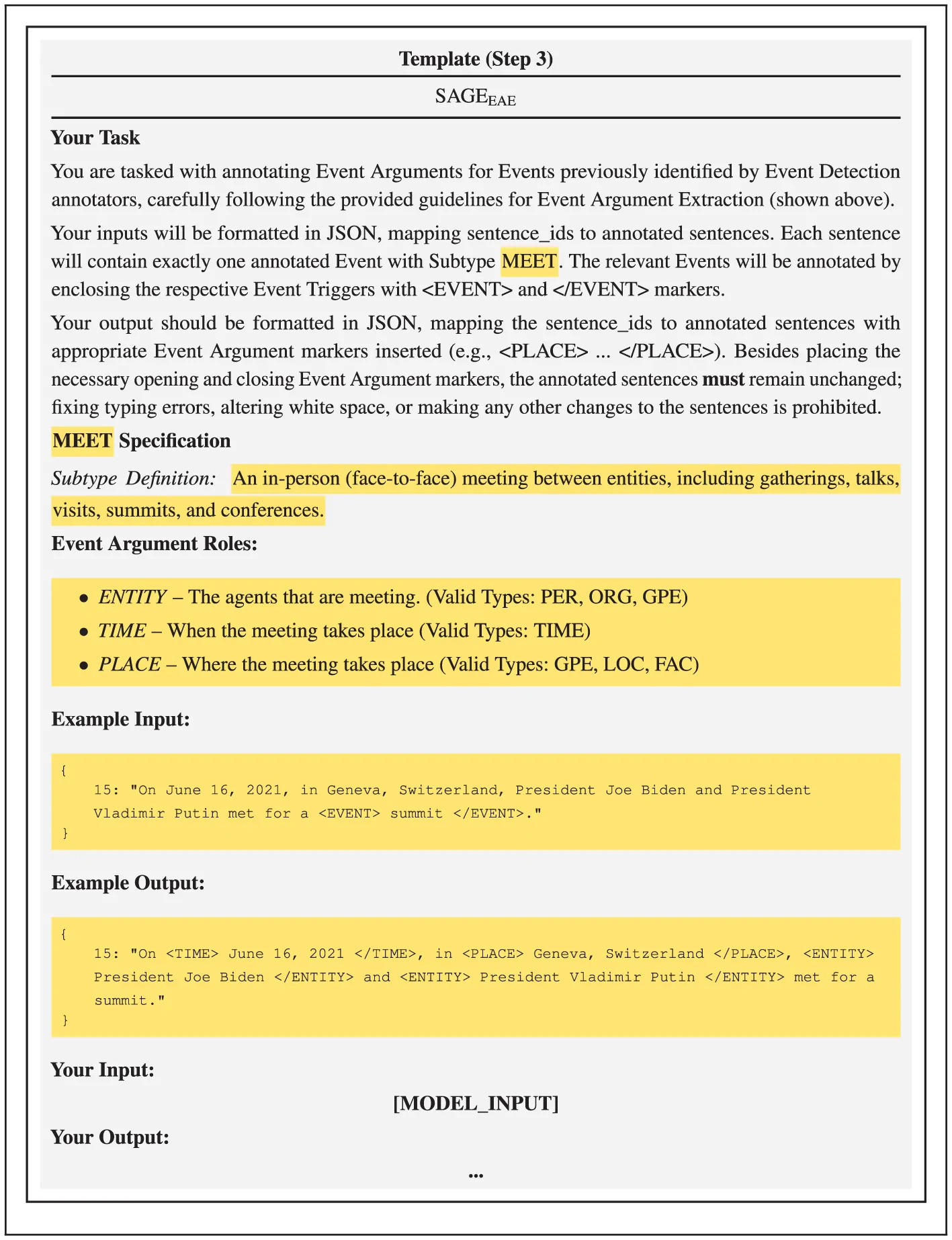
The instruction template for Step 3. The marked regions (yellow) are event subtype specific and comprise relevant context and definitions from §§6.2–6.9 Event Arguments. The examples have been generated from the *Subtype Definition* and *Event Argument Roles* using ChatGPT and demonstrate the desired output format. The [MODEL_INPUT] placeholder is replaced at runtime.

For this step, we provide the model with (1) a guideline document with detailed instructions on annotating arguments, (2) an explanation and instructions for extracting these arguments (e.g., labeled example, desired output format), and (3) the input sequence *x*_1_, ..., *x*_*n*_, modified to highlight the currently relevant event mention (*t, s*) via inline tagging. The prompt is shown in Figure 5.

**Figure 6:**
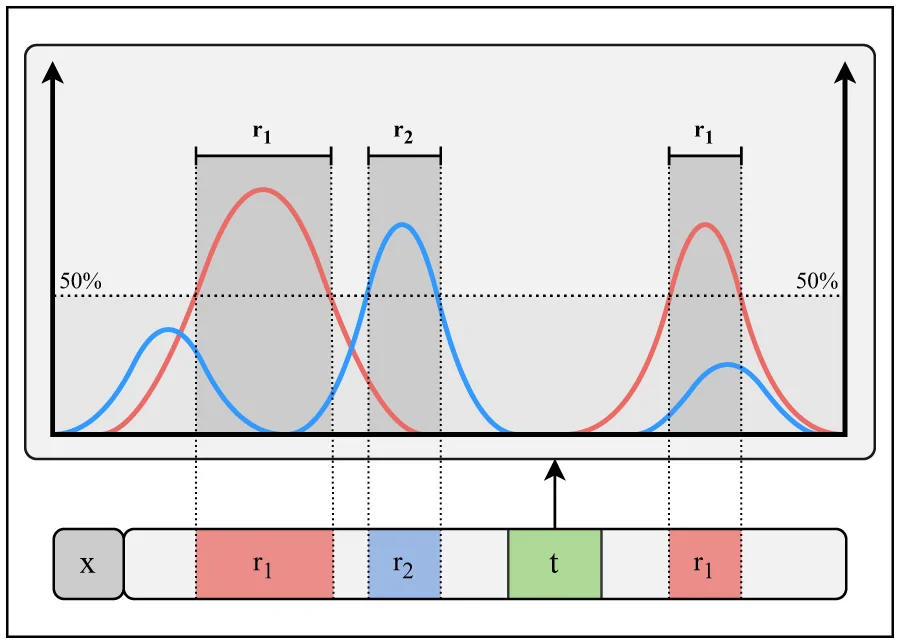
An overview of the merging process in Step 3. The event's trigger is indicated by *t* (in green). The predicted argument roles are denoted by *r*_1_ (red) and *r*_2_ (blue). The respective annotator votes $v_{i}^{r}$ (relative) are shown along the *y*-axis. Only predictions with >50% agreement are accepted.

As in Step 1 and 2, we simulate multiple annotations *A*_1_, ..., *A*_*k*_ to obtain more robust predictions. For that, the individual responses are aggregated as follows (illustrated in Figure 6):

Let $p_{1}^{r},...,p_{l}^{r}$ represent the argument spans obtained by sampling *A*_1_ through *A*_*k*_ for some (*t, s*) in $P_{2}$ and argument role $r\in R_{s}$, and let $v_{i}^{r}\in \left\{1,...,k\right\}$ denote the number of annotators *A*_1_, ..., *A*_*k*_ that predicted the token *x*_*i*_ as “representing” *r*, that is:


$$v_{i}^{r}\;:=\;|\left\{j\in \left\{1,...,l\right\}|x_{i}\text{ is covered by }p_{j}^{r}\right\}|$$

Further, we introduce $a_{i}^{r}$ := $\left({\vec{\omega }}_{i}^{r},{\vec{\omega }}_{i}^{r}\right)\in N_{x}$ to describe the *maximal* span enclosing *x*_*i*_ such that each count $v_{j}^{r}$ represents the majority of *A*_1_, ..., *A*_*k*_:


$$\begin{array}{lllll} {\vec{\omega }}_{i}^{r} & \;:=\;\min\; & \left\{j|v_{j}^{r},...,v_{i}^{r}>\frac{k}{2},j\leq i\right\}\cup \left\{i+1\right\} & \; & \;\\ {\vec{\omega }}_{i}^{r} & \;:=\;\max\; & \left\{j|v_{i}^{r},...,v_{j}^{r}>\frac{k}{2},j\geq i\right\}\cup \left\{i-1\right\} & \; & \; \end{array}$$

For an event mention (*t, s*), we obtain our final arguments predictions as follows:


$$P_{3}\;:=\;\left\{\left(t,s,a_{i}^{r},r\right)|r\in R_{s},i\in \left\{1,...,n\right\},v_{i}^{r}>\frac{k}{2}\right\}$$

## 3 Experiments

We validate our method by considering the comprehensive and well-documented *ACE 2005 English Annotation Guidelines for Events* (denoted by AGE) as a reference document. To provide more context, we proceed by outlining the AGE.

### 3.1 ACE annotation guidelines

The *ACE 2005 English Annotation Guidelines for Events*^1^ were developed particularly for annotating the ACE 2005 dataset (Walker et al., 2006). In summary, the AGE contain the following sections and contents (renamed and abridged for brevity):

- **§1 Basic Concepts**—An introduction to fundamental terminology, the underlying structures, and general concepts related to events, such as *event types, triggers*, and *arguments*.
- **§2 Annotating Event Triggers**—The guidelines for assessing the taggability of potential event mentions, covering specific word classes (e.g., verbs and nouns) and resultative-like constructions (e.g., adjectives or participles as premodifiers), conventions for handling nominal event mentions (e.g., anaphors and headless noun phrases), and guidelines for annotating complex samples (e.g., deciding between multiple possible triggers using standalone/preference rules), among other aspects.
- **§3 Event Properties**—The relevant event features further include (a) whether the event (could have) occurred (*polarity*), (b) the written (or spoken) *tense*, (c) whether the mention is generic or specific (*genericity*), and (d) whether the occurrence is expressed as if asserted or uncertain (*modality*).
- **§4 Event Coreference**—Two event mentions are coreferring if they represent the same event occurrence and involve the same underlying arguments.
- **§5 Event types**—The seven event types (e.g., *Life* and *Justice*) and 33 subtypes (e.g., *Life.Marry* and *Justice.Pardon*), described and explained using annotated example sentences, and guidelines for annotating ambiguous event mentions (e.g., *Life.Injure, Life.Die*, and *Conflict.Attack*).
- **§6.1 Annotating Event Arguments**—An introduction to event arguments (participants and general or specific attributes, such as *TIME* and *CRIME*), followed by guidelines for annotating these arguments. For instance, an argument is annotated regardless of its ‘uncertain modality' (the involvement may be hypothetical, uncertain, or speculative) *if* it cannot reasonably be interpreted as ‘uninvolved' within its respective modality (and considered event). **Note:** AGE contains no guidelines on argument boundary detection.
- **§§6.2–6.9 Event Arguments**—The potential argument roles (e.g., *Agent-ARG* and *Instrument-ARG*) with associated argument types (e.g., *Person, GPE*, and *Vehicle*) are specified for each subtype and explained via annotated sentences, with additional information on potentially confusing arguments (e.g., *Place-ARG* and *Target-ARG* in *Conflict.Attack*).

Excluding §1 Basic Concepts, the AGE document comprises an abundance of annotated examples. Note that §1 Basic Concepts, §5 Event Types, and §§6.2–6.9 Event Arguments contain the aspects normally supplied to LLMs when performing prompt-based event extraction, providing the “bare minimum” for annotation guidelines, whereas instructions that correspond to §2 Annotating Event Triggers, §3 Event Properties, and §6.1 Annotating Event Arguments are, as far as we are aware, generally neglected in the literature. These contents could, we hypothesize, improve the LLM's performance by more accurately mimicking the original annotation procedure.

### 3.2 Summarized annotation guidelines

To condense our prompts, we produce a manual summary of the AGE (which we refer to as *SAGE*). This provides more precise control over what guidelines are presented to the models, while also significantly shortening the resulting prompts (i.e., token count), which minimizes the expense incurred by closed-source LLMs. To be more specific, the AGE document is around 17,000 words, while our summary amounts to less than 3,000 words.

SAGE contains all task-relevant information described in Section 3.1, except for the content presented in §4 Coreference. To facilitate our pipeline approach, SAGE shall be split into *SAGE*_*ED*_ and *SAGE*_*EAE*_, containing the necessary guidelines for event detection and event argument extraction, respectively. SAGE_*ED*_ is required in Steps 1 and 2, while SAGE_*EAE*_ is required in Step 3.

To ensure reproducible experiments, we provide the complete SAGE_*ED*_ and SAGE_*EAE*_ documents in our Supplementary material.

### 3.3 Experimental settings

To investigate the influence of the various aspects (or *features*) of annotation guidelines, we incrementally incorporate their corresponding summarized instructions and demonstration exemplars into our guideline-based framework. This incremental procedure is conducted separately for event detection and argument extraction, following the hypothesis that progressive introduction of annotation-relevant concepts (such as properties, examples, and guidelines on boundary detection) *should* improve performance on guideline-compliant datasets if LLMs can effectively understand (and generate predictions in accordance with) such guidelines. The incremental experiments are therefore designed to isolate the contribution of each guideline component (shown below).

For event detection, we consider the following scenarios (based on SAGE_*ED*_):

- **Baseline**—incorporates the fundamental concepts from §1 Basic Concepts and descriptions for taggable event types from §5 Event Types. This scenario contains the “bare minimum” guidelines commonly found in the literature.
- **+A**—elaborates upon *Baseline* by presenting the annotation instructions on boundary detection and preference guidelines for candidate spans conveyed in §2 Annotating Event Triggers.
- **+P**—expands +*A* by providing the conventions for annotating events described in §3 Event Properties, stipulating annotation irrespective of semantic attributes such as Polarity, Tense and Modality.
- **+E**—augments +*P* by additionally including examples to demonstrate the instructions expressed in §2 Annotating Event Triggers and §3 Event Properties. Note: This scenario presents the complete SAGE_*ED*_ document to the LLM (see Supplementary material).

Likewise, we consider the following scenarios for argument extraction (based on SAGE_*EAE*_):

- **Baseline**—introduces the baseline concepts from §1 Basic Concepts and presents the required argument roles described in §§6.2–6.9 Event Arguments.
- **+A**—expands Baseline by introducing the instructions on determining (the most appropriate) event arguments from §6.1 Annotating Event Arguments. Note: Since AGE contains no guidelines on detecting *argument* boundaries, such rules are also not considered in our framework.
- **+E**—augments +*A* by including examples to demonstrate the instructions expressed in §6.1 Annotating Event Arguments. Note: This scenario employs the complete SAGE_*EAE*_ document (see Supplementary material).

However, by presenting such long guidelines to the LLM, we introduce an enormous textual overhead for only *one* sequence to be annotated, even when using our summary. To minimize this overhead, we provide the model with 16 batched samples at once whenever possible. Moreover, in Steps 2 and 3, the input samples can be batched by event types and subtypes, respectively, which avoids having to provide more than one type definitions per model request.

#### 3.3.1 Evaluation

We evaluate our guideline-based approach on event detection (after Step 2) and argument extraction (after Step 3), and condition the argument predictions on the reference (golden) event mentions as opposed to LLM-generated event mentions (from Step 2). As a consequence, our annotation framework is not evaluated end-to-end, but instead decomposed into constituent subtasks. This design choice is common in the literature (Liu et al., 2025; Sainz et al., 2024; Ma et al., 2023; Wang et al., 2023b) and is herein adopted for reasons of limited compute and, more importantly, to isolate the influence of guideline variants on model behavior. This separation avoids confounding effects caused by full-pipeline error propagation and promotes a clearer understanding of how detailed annotation guidelines can benefit LLM-generated annotations. We evaluate the subtasks as follows:

- **Event Detection**—For an input *x*, let $P_{2}\left(x\right)$ denote the predicted event mentions (from Step 2) and let $G_{2}\left(x\right)$ denote the reference event mentions. Following standard evaluation procedure, an event tuple $\left(t,s\right)\in P_{2}\left(x\right)$ is deemed correct if $\left(t,s\right)\in G_{2}\left(x\right)$. From this, we calculate the overall (micro) F1 score.
- **Argument Extraction**—-For an input *x*, let $P_{3}\left(x\right)$ denote the predicted arguments for *all* event tuples $\left(t,s\right)\in G_{2}\left(x\right)$ (use $P_{2}\left(x\right)$ for complete evaluation) and $G_{3}\left(x\right)$ represent the golden argument mentions. Again, following standard evaluation procedure, an argument prediction $\left(t,s,a,r\right)\in P_{3}\left(x\right)$, with span and subtype (*t, s*) taken for characterizing (*a, r*), is deemed correct if $\left(t,s,a,r\right)\in G_{3}\left(x\right)$. From there, we calculate the overall (micro) F1 score.

By separating both subtasks, differences in performance can be attributed more accurately to a individual guideline components (e.g., variant +A vs. variant +E) presented to the LLM, rather than unintended interactions between the different variants, such as SAGE_*ED*_ (+P) and SAGE_*EAE*_ (+E). Accordingly, performing argument extraction using reference event mentions is both sufficient and preferable for evaluating the influence of annotation guidelines on LLM performance. A notable consequence of separating these subtasks, however, is that each model's observed performance on argument extraction should be interpreted as an optimistic upper bound on its actual end-to-end performance on argument extraction in our annotation framework. Nevertheless, for existing event detection datasets in which events are annotated without corresponding argument mentions, such as GENIA (Kim et al., 2008), MAVEN (Wang et al., 2020), CySecED (Man Duc Trong et al., 2020), and FewEvent (Deng et al., 2020), assuming the existence of gold-standard event mentions is a reasonable and practically motivated scenario when aiming to extend these resources with argument annotations.

#### 3.3.2 Datasets

As our considered guideline documents SAGE_*ED*_ and SAGE_*EAE*_ have been derived directly from the *ACE 2005 English Annotation Guidelines for Events*, our experiments are naturally directed toward the corresponding ACE 2005 (Walker et al., 2006) dataset. In addition, we evaluate our approach on MEE (Pouran Ben Veyseh et al., 2022) and MAVEN (Wang et al., 2020). We choose MEE, because it features both event detection and argument extraction, and the authors declare that MEE follows the ACE 2005 event types (a subset) and annotation guidelines. While MAVEN's event schema is derived from FrameNet (Baker et al., 1998), the authors provide a (partial) mapping to convert event types between ACE 2005 and FrameNet, and further declare that MAVEN largely follows the ACE 2005 “settings and terminologies,” with no *clear* indication given about the underlying annotation guidelines.

The following describes the datasets and our pre-processing steps. An overview of the datasets is provided in Table 1.

**Table 1:** The datasets adopted for evaluating our guideline-based approach.

Dataset	Tasks	Test samples	Event schema	Guidelines
ACE 05 (W)	ED/EAE	832	ACE	ACE
ACE 05 (L)	ED/EAE	676	ACE	ACE
MEE	ED/EAE	320	ACE	ACE
MAVEN	ED	500	FrameNet	–

MAVEN's event schema (based on FrameNet) is translated into ACE's event schema through a mapping provided by MAVEN's authors Wang et al. (2020). The guidelines underlying MAVEN's annotation are not explicitly mentioned. For information on dataset pre-processing, see Section 3.3.2.

- **ACE 2005**—This dataset (licensed by the LDC) is sourced from English, Chinese, and Arabic newswire, broadcast news and conversations, and telephone speech, among others, and was annotated for various information extraction tasks. The event task includes eight event types and 33 event subtypes. We employ the default (and commonly used) pre-processing of Wadden et al. (2019)^2^ and Lin et al. (2020)^3^ and denote the processed datasets by ACE-05 (W) and ACE-05 (L). Note that Lin et al. (2020) reintroduce multi-token event triggers and consider pronouns as arguments–both of which have normally been omitted in previous works. As these changes more closely adhere to the AGE, we anticipate a marginal increase in performance on ACE-05 (L) over ACE-05 (W). For ACE-05 (W) and ACE-05 (L), we consider the entire test splits, which constitute 832 and 676 sentences (in English), respectively, to evaluate our approach.
- **MEE**—The Multilingual Event Extraction (MEE) dataset^4^ was compiled from various Wikipedia articles related to economy, politics, crime, and military, among other categories. The material spans eight different languages and the dataset contains eight event types and 16 event subtypes, offering a subset of the ACE 2005 event hierarchy. The MEE dataset includes 1,300 test samples, 1,135 of which contain one or more event mentions. However, 979 of these 1,135 samples have not been annotated with argument mentions, despite the authors claiming that MEE contains fully annotated texts for event detection and argument extraction. Upon manually checking some of the 979 partially unlabeled instances, we conclude that, based on our knowledge and understanding of the employed ACE-2005 annotation scheme, valid argument mentions are indeed present in the texts. The MEE authors Pouran Ben Veyseh et al. (2022) make no mention of this anomaly. We found that Huang et al. (2024) raised similar concerns about MEE's annotation of argument mentions. Since we discovered no additional anomalies in that regard, we evaluate our approach on the remaining 320 instances, namely the 165 samples without event mentions, and the 155 samples^5^ with proper argument mentions. We further remark that MEE annotates all coreferent mentions of an argument as argument mentions, but provides no direct information on entity coreference, which partially obstructs our (standard) evaluation for argument extraction, see Section 3.3.1. As such, it is challenging to determine how many distinct entities assume a particular argument role. To mitigate the influence of coreferring arguments on evaluation (only one mention per argument is required), we introduce the following change: For an input *x*, a reference argument $\left(t,s,a,r\right)\in G_{3}\left(x\right)$ shall be considered *false negative* if there exists no $\left(t,s,â,r\right)\in P_{3}\left(x\right)$ such that $\left(t,s,â,r\right)\in G_{3}\left(x\right)$.
- **MAVEN** – The Massive Event Detection Dataset^6^ was derived from general domain Wikipedia articles (in English). The dataset covers 168 event types primarily adopted and refined from FrameNet (Baker et al., 1998), with heuristic methods used to determine potential event mentions to be finalized by crowd-sourced annotators. The dataset contains no annotated argument mentions, but has recently been expanded to MAVEN-ARG (Wang et al., 2024). Since MAVEN does not provide any explicit event definitions, we borrowed the (partial) MAVEN-to-ACE event mapping provided by Wang et al. (2020), mapping 25 MAVEN event types onto 6 ACE event types (12 subtypes) and removing the remaining (unmapped) 143 event types. For our evaluation, we randomly select 500 samples from their validation split (no labeled test split provided) such that exactly 250 samples contain at least one ACE event—for two reasons: (a) their validation split contains 8,042 (too many) sentences, and (b) only 2,289 of these 8,042 samples contain any ACE event mentions after mapping, leaving an otherwise sparsely (< 29%) annotated evaluation split.

#### 3.3.3 Models

The experiments are conducted using these instruction-following LLMs: Llama 3.3 70B Instruct^7^, GPT-4o, and GPT-4o mini. Furthermore, we include the reasoning model o3-mini^8^.

For Llama 3.3 and GPT-4o (mini), we select a temperature of 0.3. For o3-mini, we selected the *medium* reasoning effort. Apart from limiting the number of output tokens (2^15^ for o3-mini, and 2^13^ otherwise), no additional hyperparameters were changed. In each step, we sample *k* = 5 outputs (i.e., *A*_1_, ..., *A*_*k*_) from the models and aggregate the responses accordingly. For Llama 3.3 70B Instruct, our annotation experiments took around 720 h until completion on an Nvidia A40.

To support parsing the outputs for multiple samples, we employ OpenAI's *Structured Outputs*^9^ and use *lm-format-enforcer*^10^ for Llama 3.3 to enforce specific JSON schemes in each step, mapping sentence ids to annotated sequences. For an example, see our instruction templates in Figure 5.

## 4 Results

In this section, we present a structured evaluation of our guideline-based framework, examining its performance, consistency, and practical influence for downstream model training. We pursue the following structure: First, we proceed by reporting our main results (Tables 2, 3), which showcase the influence of progressively enriched annotation guidelines for event detection and argument extraction on LLM-generated annotations. Then, to better understand performance limitations in argument extraction, we introduce a soft-matching evaluation (Section 4.2), which serves to isolate boundary detection from argument role classification. Next, we assess the faithfulness of our guideline summary (SAGE) relative to the original AGE document (Section 4.3), followed by an analysis of LLM-based guideline summaries (Section 4.4). Thereafter, we continue by comparing our results with recent prompt-based and supervised approaches (Section 4.5) to contextualize performance. Finally, we examine the practical impact our LLM-generated annotations have on downstream model training (Section 4.6) by measuring the performances under different ratios of LLM-acquired to gold-standard event annotations. Through these experiments, we seek to demonstrate the effectiveness of detailed, dataset-specific guidelines, determine key limitations, and evaluate this framework's practicality in realistic settings.

**Table 2:** The complete results for the Event Detection task.

ACE 05 (W)	GPT-4o mini			GPT-4o			o3-mini			Llama 3.3 70B		
	P	R	F	P	R	F	P	R	F	P	R	F
**Baseline**	52.4	42.3	46.8	47.7	61.8	**53.8**	63.2	46.6	53.6	32.7	53.9	**40.7**
**+ A**	51.6	45.1	48.1	46.5	63.8	**53.8**	59.4	53.7	56.4	31.3	51.9	39.1
**+ P**	54.4	44.8	49.1	45.9	63.3	53.2	63.8	57.2	**60.3**	31.2	49.4	38.3
**+ E**	53.4	46.3	**49.6**	44.5	62.8	52.1	64.9	55.7	59.9	30.0	50.4	37.6
**SOTA**	73.6 (Wang et al., 2022)											
**ACE 05 (L)**	**GPT-4o mini**			**GPT-4o**			**o3-mini**			**Llama 3.3 70B**		
	**P**	**R**	**F**	**P**	**R**	**F**	**P**	**R**	**F**	**P**	**R**	**F**
**Baseline**	58.3	41.3	48.4	53.0	62.3	**57.2**	72.1	46.6	56.6	33.6	45.4	38.6
**+ A**	57.7	43.0	49.3	49.9	62.7	55.6	70.1	56.2	**62.4**	34.5	44.5	38.9
**+ P**	58.0	42.5	49.1	48.8	61.1	54.2	71.7	53.6	61.3	35.5	43.3	39.0
**+ E**	59.6	44.7	**51.1**	50.5	63.7	56.3	67.8	53.1	59.6	34.4	46.9	**39.7**
**SOTA**	75.2 (Liu et al., 2023)											
**MEE**	**GPT-4o mini**			**GPT-4o**			**o3-mini**			**Llama 3.3 70B**		
	**P**	**R**	**F**	**P**	**R**	**F**	**P**	**R**	**F**	**P**	**R**	**F**
**Baseline**	39.4	59.2	**47.3**	40.6	64.4	**49.8**	52.4	52.4	52.4	22.2	44.8	**29.7**
**+ A**	37.9	56.9	45.4	38.2	66.0	48.4	50.5	56.0	53.1	20.9	40.8	27.6
**+ P**	37.1	58.0	45.2	38.8	64.0	48.3	50.9	55.6	**53.2**	22.4	43.6	29.6
**+ E**	36.8	59.6	45.5	38.6	62.8	47.8	49.6	56.0	52.6	21.1	44.0	28.5
**SOTA**	73.2 (Pouran Ben Veyseh et al., 2022)											
**MAVEN**	**GPT-4o mini**			**GPT-4o**			**o3-mini**			**Llama 3.3 70B**		
	**P**	**R**	**F**	**P**	**R**	**F**	**P**	**R**	**F**	**P**	**R**	**F**
**Baseline**	24.9	21.9	23.3	31.7	30.6	31.1	31.1	28.7	**29.8**	23.7	34.0	27.9
**+ A**	24.0	22.2	23.1	30.3	35.8	32.8	27.6	29.3	28.4	24.6	34.5	28.7
**+ P**	26.8	24.8	25.8	29.3	33.6	31.3	26.1	26.8	26.4	24.5	36.8	**29.4**
**+ E**	27.7	26.1	**26.9**	31.8	34.5	**33.1**	27.4	30.8	29.0	24.7	35.7	29.2
**SOTA**	N/A											

The highest F-scores are highlighted in **bold**, while Precision and Recall are underlined. Where known, we provide the supervised SOTA performance for reference.

**Table 3:** The complete results for Event Argument Extraction.

ACE 05 (W)	GPT-4o mini			GPT-4o			o3-mini			Llama 3.3 70B		
	P	R	F	P	R	F	P	R	F	P	R	F
**Baseline**	37.0	20.5	26.4	31.9	37.3	34.4	31.1	29.1	30.0	29.3	29.0	**29.1**
**+ A**	37.4	24.9	**29.9**	34.2	37.3	**35.7**	30.7	29.3	30.0	28.8	28.0	28.4
**+ E**	40.9	22.8	29.3	35.0	36.5	**35.7**	33.0	30.8	**31.9**	28.4	27.4	27.9
**SOTA**	73.5 (Hsu et al., 2022)											
**ACE 05 (L)**	**GPT-4o mini**			**GPT-4o**			**o3-mini**			**Llama 3.3 70B**		
	**P**	**R**	**F**	**P**	**R**	**F**	**P**	**R**	**F**	**P**	**R**	**F**
**Baseline**	35.5	18.8	24.5	34.3	33.4	33.8	34.8	28.6	31.4	30.1	25.7	**27.7**
**+ A**	37.5	19.5	25.6	32.8	32.4	32.6	35.3	29.5	**32.2**	29.9	25.4	27.5
**+ E**	39.8	19.7	**26.3**	35.2	33.2	**34.2**	35.3	28.7	31.7	29.5	24.7	26.9
**SOTA**	73.0 (Hsu et al., 2022)											
**MEE**	**GPT-4o mini**			**GPT-4o**			**o3-mini**			**Llama 3.3 70B**		
	**P**	**R**	**F**	**P**	**R**	**F**	**P**	**R**	**F**	**P**	**R**	**F**
**Baseline**	57.8	23.3	33.2	57.3	36.1	44.3	47.0	35.1	40.2	41.6	26.7	32.5
**+ A**	55.8	26.2	**35.7**	62.3	36.1	**45.7**	47.6	34.4	**41.5**	43.3	28.1	**34.1**
**+ E**	55.0	20.7	30.1	61.1	33.9	43.6	47.8	36.6	**41.5**	39.9	25.9	31.4
**SOTA**	69.19 (Pouran Ben Veyseh et al., 2022)											

The highest F-scores are highlighted in **bold**, while Precision and Recall are underlined. For reference, we provide the supervised SOTA performance.

### 4.1 Main results

We proceed by presenting our main results on event detection (Table 2) and argument extraction (Table 3), with supervised SOTA results for reference (Wang et al., 2022; Hsu et al., 2022; Liu et al., 2023; Pouran Ben Veyseh et al., 2022):

In Table 2, more than half of the scenarios tested (9 out of 16) benefit from more detailed SAGE_*ED*_ variants, with GPT-4o mini and o3-mini showing the highest increase in performance, gaining roughly three and six percent F1, respectively, over both ACE-05 variants. In contrast, GPT-4o and Llama 3.3 seem to profit less from more advanced SAGE_*ED*_, as they perform best when evaluated under *Baseline* in most scenarios (six out of eight). Further, while o3-mini usually lags behind GPT-4o for the *Baseline* document, o3-mini *considerably* outperforms GPT-4o on MEE and ACE-05 (both variants) by 4.9 and (up to) 7.8 points once more detailed SAGE_*ED*_ are provided.

The +A experiments boost performance over the Baseline in 8 out of 16 scenarios, five of which are related to ACE-05. The +P variants improve upon the +A variants in 7 out of 16 scenarios, which are primarily associated with Llama 3.3 and GPT-4o mini. Lastly, introducing example applications for individual rules (+E) raises prediction performance over +P variants in 8 out of 16 scenarios. However, we observe no overall pattern on improvements.

Notably, most LLMs achieve higher scores on ACE-05 (L) compared to ACE-05 (W), which is likely because the preprocessing from Lin et al. (2020) more accurately matches the AGE, see Section 3.3.2. For MEE, providing more detailed SAGE_*ED*_ reduces performance for all models except o3-mini, which may indicate a mismatch between (S)AGE and the annotation procedure used when creating MEE. Lastly, while overall scores on MAVEN increase with more detailed SAGE_*ED*_, the performance remains surprisingly low, raising doubts regarding the adequacy of the event type mapping (or considered type definitions) provided by Wang et al. (2020), see Section 3.3.2. Indeed, following a manual inspection, we observe that MAVEN contains *Attack* event instances associated with natural disasters, whereas the AGE assumes that *Attack* events are associated with people, organizations, or geopolitical entities.

In Table 3, most scenarios evaluated (8 out of 12) for event argument extraction seem to benefit from detailed annotation guidelines (+A). However, the performance improvement is less pronounced than in Table 2, permitting an increase of ≥2.0 points F-score only for GPT-4o mini. However, including demonstrations (+E) into SAGE_*EAE*_ decreases performance across most scenarios (7 out of 12), with decreases being more significant for MEE. Further, for ACE-05 (either variant), even our highest scores fall below 50% of the supervised SOTA, whereas the highest scores for MEE yield around 66% of the SOTA, which is likely caused by our opportunistic evaluation for MEE, see Section 3.3.2.

### 4.2 Results with soft matching

We observe that results on argument extraction, despite the gained improvements in performance from more procedural task guidelines, remain substantially behind the supervised SOTA performance, matching prior related works (Li et al., 2023; Pang et al., 2023; Chen et al., 2024; Han et al., 2024). As previously discussed, in contrast to the event detection guidelines, the argument extraction guidelines contain no specific instructions on detecting an argument's boundaries. Therefore, to evaluate whether the observed deviation to SOTA performance is primarily related to argument *boundary detection* rather than argument identification and classification, we introduce a span-based soft-matching evaluation.

In this relaxed evaluation, a predicted argument span and role $\left(â,\hat{r}\right)$ for an event (*t, s*) is considered *correct* if there exists a golden labeled argument (*a, r*) for the event (*t, s*), such that $\hat{r}=r$ and â⊆*a* (i.e., â is completely contained in *a*, as opposed to â = *a*). Thereby, we can measure whether the LLM correctly identifies the argument's role and approximate position. The results are provided in Table 4.

**Table 4:** The results for Event Argument Extraction when evaluated by employing our soft-matching strategy.

ACE 05 (W)	GPT-4o mini			GPT-4o			o3-mini			Llama 3.3 70B		
	P	R	F	P	R	F	P	R	F	P	R	F
**Baseline**	57.6	31.9	41.1	52.4	63.6	57.5	72.9	71.5	**72.1**	43.1	42.8	**42.9**
**+ A**	54.6	36.3	**43.7**	54.7	61.9	**58.1**	69.8	69.8	69.8	43.3	42.0	42.6
**+ E**	61.0	34.0	43.6	55.7	59.9	57.7	71.5	70.0	70.8	43.4	42.0	42.7
**SOTA**	73.5 (Hsu et al., 2022)											
**ACE 05 (L)**	**GPT-4o mini**			**GPT-4o**			**o3-mini**			**Llama 3.3 70B**		
	**P**	**R**	**F**	**P**	**R**	**F**	**P**	**R**	**F**	**P**	**R**	**F**
**Baseline**	56.6	29.9	39.1	56.8	57.0	**56.9**	76.0	65.6	70.4	43.6	37.2	40.2
**+ A**	57.8	30.1	39.6	55.4	56.4	55.9	76.0	66.3	**70.8**	45.2	38.4	**41.5**
**+ E**	39.8	31.5	**42.2**	57.6	55.8	56.7	75.7	64.8	69.8	44.6	37.5	40.8
**SOTA**	73.0 (Hsu et al., 2022)											
**MEE**	**GPT-4o mini**			**GPT-4o**			**o3-mini**			**Llama 3.3 70B**		
	**P**	**R**	**F**	**P**	**R**	**F**	**P**	**R**	**F**	**P**	**R**	**F**
**Baseline**	69.8	28.6	40.5	75.8	47.6	58.5	81.2	60.4	69.3	55.5	35.6	43.4
**+ A**	68.0	32.1	**43.6**	82.9	44.7	**60.6**	82.4	59.3	69.0	55.8	36.2	**43.9**
**+ E**	67.5	25.8	37.3	83.2	45.7	59.0	82.6	63.1	**71.5**	52.6	34.1	41.4
**SOTA**	69.19 (Pouran Ben Veyseh et al., 2022)											

The highest F-scores are highlighted in **bold**, while Precision and Recall are underlined. The supervised SOTA scores (obtained using *strict* evaluation) are reported for reference.

While exhibiting trends similar to the standard (strict) evaluation in Table 3, overall results are substantially improved for all scenarios, achieving up to 72.1 and 70.8 percent F1 for ACE-05 (W) and ACE-05 (L), and 71.5 percent F1 for MEE. Notably, the strongest results are achieved by o3-mini, surpassing GPT-4o by more than 10 percent F1 on all datasets, trailing only 1.4 and 2.2 percent F1 behind SOTA scores for ACE-05 (W) and ACE-05 (L). Of course, these metrics are not directly comparable, as SOTA results are calculated using a standard strict evaluation, but they highlight an important limitation (and remaining challenge) of our approach:

These results indicate that the primary limitation of prompt-based argument extraction is related to precise boundary detection, not semantic argument role identification. Considering how only argument boundary matching is relaxed, and that our employed argument guidelines (SAGE_*EAE*_) contain no information on argument boundaries, the results of our relaxed evaluation suggest that prompt-based argument extraction could be improved via additional, boundary-aware instructions. Moreover, as arguments are primarily represented by mentions of entities, values, and temporal expressions, such boundary-aware guidelines could prove beneficial for detecting entity mentions, extending beyond argument extraction.

### 4.3 Results with complete ACE 2005 guidelines

Since our experiments are conducted using SAGE, a condensed version of the original ACE 2005 annotation guidelines (AGE), it is important to assess the extent to which this summary (or compression) may influence the evaluation outcomes compared to experiments performed with the complete AGE document. In the following, we, therefore, briefly evaluate the faithfulness (i.e., adherence) of SAGE with respect to AGE though further experiments.

To evaluate the faithfulness of SAGE with respect to AGE, we present the results obtained on ACE-05 (W) by presenting the original AGE document to o3-mini (setting *k* = 5). For event detection and argument extraction, we replicate our baseline, +A, and +P experiments by successively including the respective AGE sections, as presented in Section 3.1 and described in Section 3. Notably, the AGE sections already contain various examples that correspond to SAGE with +E variants. Hence, without rewriting the original document, there exists no straightforward +P variant. We conduct any comparisons between SAGE- and AGE-based experiments accordingly. The results are presented in Table 5.

**Table 5:** The SAGE- and AGE-based results for Event Detection and Event Argument Extraction.

ACE-05 (W) o3-mini	SAGE						AGE					
	ED			EAE			ED			EAE		
	P	R	F	P	R	F	P	R	F	P	R	F
**Baseline**	63.2	46.6	53.6	31.1	29.1	30.0	56.2	51.6	53.8	30.6	28.1	29.3
**+ A**	59.4	53.7	56.4	30.7	29.3	30.0	55.0	59.0	56.9	↓	↓	↓
**+ P**	63.8	57.2	**60.3**	–	–	–	↓	↓	↓	–	–	–
**+ E**	64.9	55.7	59.9	33.0	33.0	**31.9**	53.4	59.2	56.2	31.6	29.5	**30.5**

The highest F-scores are highlighted in **bold**. The vertical ↓ arrows mark results which have instead been attributed to the +E experiments, because the AGE already contain many examples that would otherwise be introduced through the +E modifier. The underlined results correspond to experiments with no SAGE-based equivalent (due to the inherent +E modifier in AGE), as no SAGE-based experiment with *only* the +A and +E modifiers are considered.

For the baseline and +A experiments, the SAGE- and AGE-based results are no more than 0.7 score points apart. AGE surpasses SAGE on event detection, while SAGE outperforms AGE on argument extraction. However, when comparing results for the complete SAGE and AGE documents (+E), SAGE outperforms AGE by 3.7 and 1.4 points on event detection and argument extraction. In terms of utility, SAGE is comparable to AGE.

### 4.4 Results with automatic guideline summaries

Since SAGE constitutes a manually constructed summary of the original ACE 2005 annotation guidelines (AGE), we further seek to assess whether such manual compression (i.e., human supervision) is necessary, or whether comparable results are obtained from LLM-generated summaries. In the following, we therefore briefly evaluate the effectiveness of automatically generated AGE summaries through additional experiments.

**Figure 7:**
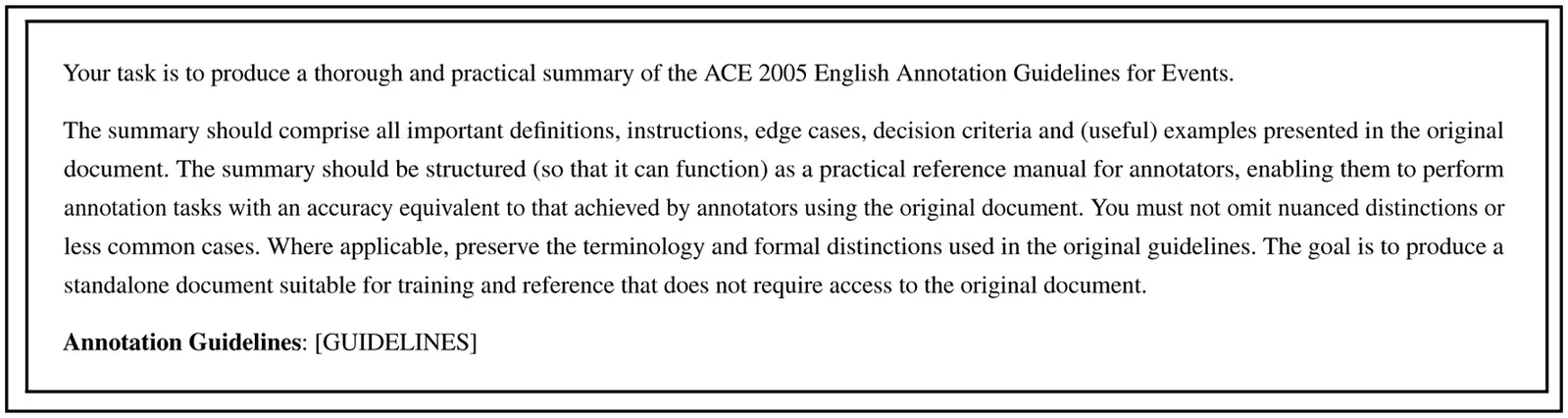
The prompt (template) for generating the LAGE^(*i*)^ documents, summarizing AGE.

We substitute our SAGE documents with LLM-generated AGE summaries and compare the experimental results. Concretely, we prompt GPT-4.1 with standard hyperparameters as the summarizing LLM to generate three different AGE summaries ${\text{LAGE}}_{\text{ED}}^{\left(i\right)}$ and ${\text{LAGE}}_{\text{EAE}}^{\left(i\right)}$, with *i*∈{1, 2, 3}. The prompt used is provided in Figure 7. Notably, LAGE corresponds to SAGE with +E modifiers. We evaluate our approach for each of the LAGE^(*i*)^ documents, using o3-mini and GPT-4o as annotators. The results are reported in Table 6.

**Table 6:** The results (mean scores) obtained via LAGE with GPT-4o and o3-mini as annotating LLMs.

ED	GPT-4o					o3-mini				
	P	R	F	LAGE^*^	SAGE^(*)^	P	R	F	LAGE^*^	SAGE^(*)^
**ACE-05 (W)**	43.0	68.4	52.9	53.4	52.1 (53.8)	54.8	53.4	53.8	55.0	59.9 (60.3)
**ACE-05 (L)**	47.4	65.7	55.0	56.7	56.3 (57.2)	58.0	57.2	57.6	59.4	59.6 (62.4)
**MEE**	36.6	63.2	46.3	47.3	47.8 (49.8)	45.1	62.9	52.5	53.7	52.6 (53.2)
**MAVEN**	26.5	33.2	29.5	30.6	33.1 (33.1)	24.9	30.2	27.3	27.3	29.0 (29.8)
**EAE (strict)**	**GPT-4o**					**o3-mini**				
	**P**	**R**	**F**	**LAGE** ^*^	**SAGE** ^(*)^	**P**	**R**	**F**	**LAGE** ^*^	**SAGE** ^(*)^
**ACE-05 (W)**	36.0	34.7	35.3	36.9	35.7 (35.7)	32.8	31.0	31.9	33.6	31.9 (31.9)
**ACE-05 (L)**	34.9	31.0	32.8	33.3	34.2 (34.2)	36.0	30.3	32.9	34.4	31.7 (32.3)
**MEE**	67.2	33.2	44.4	47.0	43.6 (45.7)	48.6	36.1	41.4	41.9	41.5 (41.5)
**EAE (soft)**	**GPT-4o**					**o3-mini**				
	**P**	**R**	**F**	**LAGE** ^*^	**SAGE** ^(*)^	**P**	**R**	**F**	**LAGE** ^*^	**SAGE** ^(*)^
**ACE-05 (W)**	59.1	58.6	58.8	59.9	57.7 (58.1)	70.9	69.9	70.4	70.9	70.8 (72.1)
**ACE-05 (L)**	58.2	52.7	55.3	56.2	56.7 (56.9)	75.3	66.0	70.3	70.7	69.8 (70.8)
**MEE**	85.5	42.2	56.4	59.8	59.0 (60.6)	81.5	59.9	69.1	71.4	71.5 (71.5)

The LAGE^*^ columns describe the highest F-scores observed using LAGE, and the SAGE^(*)^ columns describe the corresponding SAGE (+E variants) F-scores, as well as the *overall* highest SAGE (any variant) F-scores in parentheses.

On event detection, LAGE is generally surpassed by SAGE, with performance decreasing by (up to) 5.7 points with o3-mini on ACE-05 (W). However, LAGE is competitive to SAGE for argument extraction, showcasing that LLM-based summaries can indeed be feasible options for that subtask.

### 4.5 Comparison with existing methods

In Table 7, we compare our results with various prompt-tuning and ICL methods studied in recent works. We examine the following methods:

**Table 7:** A comparison with supervised and ICL-focused approaches (in F-score).

Method	Setting	ACE-05 (W)		ACE-05 (L)	
		ED	EAE	ED	EAE
**GoLLIE**	tuned	–	–	72.2	68.6
**PEE**	tuned	72.6	69.2	72.6	69.2
**Guide 100**	tuned	37.1	–	37.1	–
**Guide 2k**	tuned	60.3	–	60.3	–
**PEE**	0-shot	13.2	30.4	13.2	30.4
**GPT-IE**	5-shot	47.3 (45.9)	34.4 (41.2)	47.2	36.3
Code4	0-shot	–	–	–	37.8†
Code4	20-shot	–	–	–	60.4†
**Ours**	1-shot	60.3	35.7 (72.2)	62.4	34.2 (70.8)

The results for soft-matching evaluations are presented in parenthesis. The dagger (†) denotes results with non-standard evaluations.

- Code4**Struct** (Wang et al., 2023a)—A code-oriented approach that employs baseline guidelines, validated on EAE. The results are reported for zero- and 20-shot scenarios. The evaluation is performed after post-processing the predictions using spaCy.
- **GoLLIE** (Sainz et al., 2024)—An LLM trained to comply with (baseline) guidelines. The evaluation on ACE 2005 was performed fully supervised.
- Guide**line** (Srivastava et al., 2025)—An approach trained on various human- and LLM-written guidelines. We include the overall best results for training on 100 and 2,000 samples.
- **GPT-IE** (Han et al., 2024)—An extensive study on LLM's (GPT-4) information extraction capabilities. We present their highest F-scores, including the soft-matching results.
- **LLM-**PEE (Liu et al., 2025)—An LLM-based pipeline that employs (baseline) instructions for the extraction. The results for ACE 2005 are reported for the supervised and zero-shot settings.

Notably, Sainz et al. (2024), Srivastava et al. (2025), Liu et al. (2025), and Wang et al. (2023a) conduct the extraction code generation, whereas our approach annotates the desired information, such as event spans and arguments, directly within the input sequence.

For event detection, where extensive, boundary-aware annotation guidelines are available, our guideline approach substantially outperforms the considered ICL methods, markedly narrowing the gap to SOTA supervised approaches such as GoLLIE and PEE. However, for argument extraction, our guideline approach obtains only average performance and is outperformed by few-shot ICL methods such as Code4Struct. As discussed in Section 4.2, we suppose that additional guidelines on boundary detection could provide notable improvements for argument extraction.

### 4.6 Training on LLM-generated annotations

While the previous experiments evaluate our LLM-generated annotations directly, a likewise important question is whether these labels are suitable for training downstream models, distilling the LLM-based predictions into specialized and computationally less expensive models. Therefore, we examine the practical viability of LLM-generated annotations in a supervised scenario.

We evaluate the practical viability of these LLM-generated annotations by training various supervised baselines with annotations of varying degrees of syntheticity, using MEE and ACE 2005 as our benchmarks. For that, we annotate both datasets using our pipeline. Based on our previous results, the annotations are generated with o3-mini as the annotating LLM and are supplemented with SAGE_*ED*_ (+P) and SAGE_*EAE*_ (+E) as the annotation guidelines. The annotation procedures are conducted in accordance with Section 3.3. For dataset processing and model training,^11^ we employ TextEE (Huang et al., 2024), a standardized and reproducible benchmark for training and evaluating event extraction methods. The considered baselines are RoBERTa+CRF (Liu et al., 2019), EEQA (Du and Cardie, 2020), DEGREE (Hsu et al., 2022), and TagPrime (Hsu et al., 2023), because they support training for event detection and argument extraction separately and require no further annotations (e.g., entities).

We examine the influence of increasing annotation syntheticity by substituting the original annotations of ⌊α*n*⌋ (where α∈[0, 1] and *n* the number of training samples) random training samples with LLM-generated annotations for each of the five dataset splits proposed by TextEE. The subtasks event detection and argument extraction are evaluated separately, following Section 3.3. However, the ambiguous annotation of MEE prevents an adequate training and evaluation under argument extraction, see Section 3.3.2. We contrast these experiments on the α annotation syntheticity by training each baseline on the remaining *n*−⌊α*n*⌋ golden training samples, without label substitutions. Thereby, one can observe whether introducing LLM-generated annotations into an existing dataset can improve the baseline results. Overall, the experiments took around 1,600 GPU-h on an Nvidia A40.

The joined results, which include performance comparisons to training on (purely) human labeled samples as well as our soft-matching approach for argument extraction, are presented in Table 8. The experiments demonstrate that increasing the syntheticity in human expert labeled datasets consistently decreases the baseline's performance, showing a steady (even growing) decline as α increases. Of course, this outcome is to be expected because our pipelined approach is generally surpassed in performance by the considered SOTA baselines (α = 0). Likewise, comparing the resulting performance for α∈(0, 1) with that achieved by training purely on the remaining *n*−⌊α*n*⌋ golden labeled samples, we observe how employing such LLM-generated annotations always decreases (↓) model performance.

**Table 8:** The results (mean F-score) for baseline training for varying degrees of α.

Event detection								
**ACE 2005**	α = 0	$\alpha =\frac{1}{4}$		$\alpha =\frac{1}{2}$		$\alpha =\frac{3}{4}$		α = 1
**RoBERTa**	70.3	68.2	2.1↓	66.7	2.6↓	61.9	6.1↓	57.0
**EEQA**	71.9	69.7	2.8↓	68.2	2.4↓	64.2	6.9↓	60.4
**Degree**	69.3	66.0	0.6↓	64.5	2.6↓	61.2	4.7↓	57.7
**TagPrime**	69.2	67.3	1.6↓	65.8	2.8↓	61.6	6.2↓	55.8
**MEE**	α = 0	$\alpha =\frac{1}{4}$		$\alpha =\frac{1}{2}$		$\alpha =\frac{3}{4}$		α = 1
**RoBERTa**	80.5	77.4	2.5↓	73.0	5.5↓	67.3	10.4↓	61.4
**EEQA**	80.4	78.0	1.9↓	74.0	5.0↓	67.5	9.8↓	63.0
**Degree**	79.5	77.0	1.4↓	73.0	4.9↓	68.8	7.1↓	62.6
**TagPrime**	80.6	77.6	2.5↓	73.7	5.3↓	68.3	9.5↓	62.3
**Event Argument Extraction**								
**ACE 2005 (strict)**	α = 0	$\alpha =\frac{1}{4}$		$\alpha =\frac{1}{2}$		$\alpha =\frac{3}{4}$		α = 1
**RoBERTa**	72.5	67.9	3.3↓	57.1	12.0↓	46.0	15.4↓	32.7
**EEQA**	70.7	64.6	5.5↓	59.4	9.0↓	53.2	13.9↓	44.2
**Degree**	72.6	66.0	5.1↓	57.3	12.8↓	47.1	18.5↓	34.7
**TagPrime**	74.5	70.5	3.7↓	62.0	11.5↓	46.8	22.0↓	34.5
**ACE 2005 (soft)**	α = 0	$\alpha =\frac{1}{4}$		$\alpha =\frac{1}{2}$		$\alpha =\frac{3}{4}$		α = 1
**RoBERTa**	73.3	71.5	1.0↓	69.8	0.3↓	68.1	4.7↑	67.2
**EEQA**	75.5	77.5	2.1↑	75.3	1.8↑	73.4	1.0↓	69.6
**Degree**	73.9	72.5	0.3↑	70.4	0.7↓	69.8	1.7↑	69.0
**TagPrime**	75.8	74.4	0.7↓	72.9	2.1↓	71.4	0.3↑	69.8

The impact (delta) of including ⌈α*n*⌉ LLM-generated annotations over using (only) *n*−⌊α*n*⌋ golden samples is marked as positive (↑) or negative (↓).

However, when evaluated with soft-matching, our LLM-generated annotations are beneficial (↑) for 6 out of 12 scenarios, even surpassing the SOTA (α = 0) by 2.0 F-points for EEQA. Further, when changing α from 0 to 1, results only decrease by 5.7 F-points on average, demonstrating consistent performance. These findings indicate that, while LLM-generated event annotations are not (yet) a substitute for human-labeled samples in supervised scenarios, they provide a notable learning signal under relaxed settings and could serve to supplement supervision in low-resource scenarios.

## 5 Discussion

In summary, our experiments demonstrate how introducing detailed, task-specific annotation guidelines into LLM prompts can strongly increase the alignment between LLM-generated and gold-standard annotations *if* the provided guideline document and datasets adopted for evaluation are *sufficiently compatible*. For event extraction, we showcase that general-purpose LLMs require (and clearly benefit from) prescriptive, fine-grained instructions that encompass essential annotation concepts and decision boundaries, without leaving such aspects open to (subjective) interpretation–unless requested otherwise (Röttger et al., 2022).

Under this assumption, and adopting the ACE 2005 event annotation guidelines as a reference document, we achieved an increase in event detection performance by nearly seven percent F1 on the ACE 2005 dataset (Walker et al., 2006), *without* requiring a labeled dataset for example selection as normally assumed in related prompt-centric studies (Wang et al., 2023a; Srivastava et al., 2025).

Following the same argument, by conducting our evaluation on datasets that are *less* guideline-compliant, performance is expected to decrease as instructions become more specific. Indeed, this behavior is observed with both MEE and MAVEN which, although reportedly in adherence to ACE 2005, exhibit a notable discrepancy to ACE 2005, including an incomplete (and incorrect) mapping of event types between FrameNet and ACE across MAVEN, and ignorance of guidelines on argument preference in MEE, see Section 3.3.2. For event detection, these datasets show a significantly reduced average performance and a considerably reduced increase (even decrease, for MEE) in performance, whereas ACE-05 (W) and ACE-05 (L), following the inherently closer alignment with ACE 2005, yield more consistent improvements, especially for the reasoning LLM o3-mini.

Overall, providing richer SAGE documents has proven markedly more beneficial for annotating event mentions than extracting event arguments, where performance has increased only marginally. However, following a soft-matching evaluation in Section 4.2, where we decouple precise argument identification from semantic argument role classification, our findings indicate that *accurate* argument boundary detection poses an important remaining challenge, with relaxed argument detection and classification reaching near SOTA performance. We propose two possible reasons for the imprecise argument boundary detection. First, the method through which model responses are aggregated (see Figure 6), favoring a medial phrase within the argument. Second, the absence of annotation guidelines on argument boundary detection in AGE (and therefore in SAGE). These results further emphasize the potential advantages of incorporating more task specific annotation guidelines when using LLMs to generate synthetic datasets or annotations. Therefore, we propose to integrate the original *Entity, Value*, and *TIMEX* annotation guidelines^12^ into SAGE_*EAE*_ to improve the results on argument extraction.

In Section 4.6, we further applied our approach to annotate MEE and ACE 2005 and used the resulting datasets for training multiple SOTA baselines. Our findings suggest that (a) training on such annotations when no labeled samples are available provides a strong baseline, and (b) enriching existing datasets with LLM-generated argument annotations can markedly improve results when evaluated with soft-matching, surpassing even SOTA scores.

Nevertheless, our guideline-based approach performs considerably below supervised SOTA, trailing by approximately 13 percent F1 for event detection (see Table 2) and 42 percent F1 for (strict) argument extraction (see Table 3) on ACE 2005. This discrepancy in performance can be attributed to the underlying conceptual differences between these two paradigms. For instance, supervised approaches benefit from task-specific optimization over labeled datasets with gold-standard annotations, allowing these methods to (implicitly) internalize the annotation guidelines underlying the dataset's creation, including more sublime decision boundaries, such as preferring nouns over verbs as event triggers, or biases introduced during corpus preprocessing, see ACE-05 (W) and ACE-05 (L) in Section 3.3.2. In contrast, our guideline-based approach, where no labeled dataset is assumed for training, instructs LLMs to generate (or reproduce) said gold-standard annotations *from* such annotation guidelines, demanding an instruction following proficiency (and consistency) on par with human experts. This challenge is further exacerbated when the considered guidelines are *incomplete*, omitting essential concepts or instructions such as rules on argument boundary detection, as demonstrated by our relaxed argument boundary evaluation in Section 4.2. In these cases, where an evaluation-relevant annotation concept remains underspecified (or is omitted entirely), the resulting performance depends strongly on an LLM's internal bias regarding that concept, and therefore conflates an “accidental alignment” between the model's standard behavior and the dataset's gold-standard annotation with the LLM's *true* understanding of the task.

### 5.1 Related work

#### 5.1.1 LLMs as annotators

Recent studies have increasingly investigated pre-trained LLMs as automated annotators for NLP datasets, examining their usefulness, effectiveness, robustness, and agreement with trained, human annotators across a plethora of labeling tasks (Wang et al., 2021; Ding et al., 2023; Gilardi et al., 2023; Törnberg, 2023; Li, 2024; Tan et al., 2024; Lindahl, 2025; Calderon et al., 2025).

Notably, recent works showcased that LLM-based annotations (or predictions in general) can be enhanced substantially when LLMs are presented with well-structured and -formulated instructions (Koike et al., 2024; Long et al., 2025; Ngweta et al., 2025), explicit annotation criteria (Zhao et al., 2023; Peskine et al., 2023; Zhong et al., 2026), and in-context learning demonstrations (Aldeen et al., 2023; He et al., 2024; Das et al., 2024). In particular, He et al. (2024) showcase that employing an *explain-then-annotate* approach improves performance and consistency of LLM annotations, with further studies reporting that LLM-based annotation can be competitive with (and outperform) crowd-sourced annotations at considerably reduced cost and annotation time (Gilardi et al., 2023; Törnberg, 2023; Horych et al., 2025; Calderon et al., 2025; Kasner et al., 2026).

LLMs have demonstrated promising performance on straightforward tasks such as sequence classification (Törnberg, 2023; Gilardi et al., 2023; Nasution and Onan, 2024; Ahmed et al., 2024; Ma et al., 2025; Horych et al., 2025; Zhang et al., 2026), question answering (He et al., 2024; Schmidt et al., 2024; Radevski et al., 2025), named entity detection (Dao et al., 2025; Bai et al., 2025; Wang et al., 2025), and low-resource or long-tailed labeling scenarios (Kan et al., 2024; Nasution and Onan, 2024; Chen et al., 2024; Dao et al., 2025; Jadhav et al., 2025).

However, performance is substantially decreased when pretrained LLMs are required to perform more complex tasks such as structured event extraction (Pang et al., 2023; Gao et al., 2023; Chen et al., 2024), where proper annotation often involves adherence to extensive guidelines on trigger identification, argument classification, boundary detection and distinctions (or preferences) between semantically similar (or co-referent) triggers and arguments. For these tasks, human annotation is therefore frequently supported by *extensive* manuals (i.e., annotation guidelines) that introduce and demonstrate the required decision rules and concepts, and which are typically *not* provided to LLMs during inference.

#### 5.1.2 In-context learning for event extraction

Recent works on prompt-based event extraction (Gao et al., 2023; Ma et al., 2023; Li et al., 2023; Pang et al., 2023; Wei et al., 2024; Chen et al., 2024; Han et al., 2024) have explored the capacity of generative pretrained LLMs, such as GPT-4 (OpenAI, 2024) and PaLM (Chowdhery et al., 2024), for structured event extraction and comparable tasks. These works consistently demonstrate that – despite advances on few-shot methods (Yang et al., 2023; Shiri et al., 2024; Gedeon, 2025; Bonisoli et al., 2025), definition-enhanced prompting (Liu and Luo, 2024; Cai et al., 2024; Sun and Xiao, 2024; Sainz et al., 2024; Bonisoli et al., 2025; Srivastava et al., 2025), and self-refinement or -consistency approaches (Pang et al., 2023; Zhang et al., 2024)—LLM performance remains substantially below supervised, fine-tuned extraction approaches, for example, DEGREE (Hsu et al., 2022) and RexUIE (Liu et al., 2023).

These works can largely be categorized as *example*- or *definition*-driven approaches, with overlaps between these categories. The example-driven methods, such as Yang et al. (2023), Shiri et al. (2024), Gedeon (2025), and Bonisoli et al. (2025), are primarily occupied with providing labeled examples to LLMs as demonstrations from which the model is expected to generalize, normally requiring a labeled dataset for sampling these examples. In contrast, definition- or “guideline”-driven methods, such as Chen et al. (2020), Pang et al. (2023), Sun and Xiao (2024), Sainz et al. (2024), and Srivastava et al. (2025), seek to generalize from more exhaustive task descriptions, augmenting model prompts with task-related information such as label definitions or hierarchical schema information.

However, across these works, prompts are primarily designed from lightweight, schema-level instructions, commonly consisting of a task description, event schemas, target labels with brief label descriptions, and a limited number of exemplars for demonstration. While these aspects provide information on *what* to annotate, they seldom contain instructions on *how*, thereby omitting crucial procedural annotation criteria commonly provided to human annotators via annotation guidelines, including guidance on ambiguity resolution, negative constraints, and boundary detection.

For instance, Gao et al. (2023) investigate ChatGPT's feasibility for event detection on ACE 2005 (Walker et al., 2006). Their prompts entailed (1) a brief task description that introduces events and event triggers, (2) event types with definitions, and (3) positive and negative demonstrations. Although ChatGPT is substantially outperformed by supervised approaches, the ensuing ablation suggests that continually introducing new sources of task-related guidance consistently increases performance. Likewise, Wei et al. (2024) and Han et al. (2024) evaluate GPT-3.5 Turbo and GPT-4's capacities for information extraction, including event extraction. The models are primarily provided with definitions on event types and argument roles, without more detailed task-relevant instructions. On ACE 2005, both methods are substantially outperformed by supervised approaches.

We recognize analogous prompting or in-context learning methods are conducted by Chen et al. (2020), Wang et al. (2023a), Li et al. (2023), Liu and Luo (2024), and Chen et al. (2024).

Notably, Pang et al. (2023) employ LLMs to extract the underlying annotation guidelines from annotated sentences to support inference, and while the precise format of these extracted rules is unclear, the examples presented in their case study show that guidelines generated mainly involve label type definitions and demonstrations. Similarly, Sainz et al. (2024) and Srivastava et al. (2025) claim to adopt “annotation guidelines” to improve LLM outputs by following the paradigm of supervised instruction-tuning. However, Sainz et al. (2024) assimilate only definitions on event types, schemas, and arguments, with no additional task-related instructions, and Srivastava et al. (2025) likewise integrate only information that aids in demonstrating, explaining, or distinguishing event types and argument roles, with no underlying guidelines that generalize beyond these specific labels. We observe a notable exception in Sun and Xiao (2024), where guidance on the syntactic structure of arguments is provided, specifying that they may appear as named entities, pronouns, or noun, verb, adjective, and adverb phrases.

These works showcase that enriched task guidance can improve an LLM's extractive capability in zero-shot and few-shot scenarios, yet they do not investigate the influence of exhaustive annotation guidelines comparable to those given to human annotators. Thus motivated, we examine how adopting *detailed* annotation guidelines can influence in-context learning performance.

### 5.2 Limitations

Although our guideline-driven approach attempts to minimize the need for manual, human expert annotations, our method *still* requires (very) extensive annotation guidelines (e.g., manually designed) and granular event type and argument role descriptions.

Also, during a dataset's creation, event and argument mentions are (normally) annotated throughout entire paragraphs or documents (at once), whereas our approach only considers sentence-based event extraction due to dataset pre-processing.

Furthermore, without knowing how “faithful” the annotation references are toward (S)AGE, one cannot make definitive claims regarding the overall quality (i.e., usefulness) of any LLM-generated, synthetic event annotations. For instance, should the ACE-05 (W) annotation references deviate too significantly from (S)AGE, then considering their “golden” event and argument mentions is potentially unsuitable for evaluating such LLM annotations—especially when generated with SAGE. To address this, future works could directly compare reference with LLM-generated annotations and thereby provide a more practical analysis of their respective properties.

Additionally, our aggregation procedures, most notably that introduced in Step 1, have implications worth mentioning. Obviously, any accurate event mention that is rejected (or changed) by merging the outputs of *A*_1_, ..., *A*_*k*_ in Step 1 cannot be considered for subsequent annotations, thereby reducing the overall Recall achieved by this approach. While this scenario could be addressed by forwarding *all* unique event triggers to Step 2, thus postponing our candidate selection until some (preferably the false positive) predictions are classified to be *NOTA*, this would significantly increase the number of requests to be handled by the LLM.

Besides, our approach is (currently) incapable of detecting and removing (or combining) semantically identical predictions where two events are co-referent (§4, AGE), and one of the two triggers violates (S)AGE. For example, consider the sentence “*they paid the fine*.” Here, both *paid* and *fine* can reasonably be considered to represent the FINE event. In this instance, the *Stand-Alone Noun Rule* (§2, SAGE) dictates that *fine* (the noun) should be the preferred event trigger. Our approach, however, may forward both *paid* and *fine* as candidate mentions to Step 2, potentially producing two semantically identical predictions, thus lowering Precision.

Moreover, from a technical standpoint, following the (unmodified) use of conversational LLMs, our approach may demonstrate some unwanted behavior (as opposed to classical sequence labeling methods) when inserting markers into some input *x*, because the LLM is not *technically* prohibited from otherwise modifying the sequence, for example, fixing typing errors or altering white space. In extreme cases, the output may become altered beyond recognition, requiring another attempt on annotation. Of course, these scenarios can easily be avoided by employing constrained decoding strategies that, in each time step, only permit the LLM to produce the next input token in *x* or a (valid) marker.

Regarding Section 4.6, we find that employing our pipelined approach *as-is* to supplement an existing dataset that already contains expert labeled samples is not advisable (unless argument extraction with fuzzy boundaries is acceptable). Instead, we consider it more suitable to provide a solid baseline when labeled samples are scarce. Should, however, a strong selection of labeled samples be available (which our work does not assume), then it might be worth employing both annotation guidelines *and* approaches for selecting ICL examples to (further) improve our pipeline's annotations. In addition, we note that our evaluation only considers annotations from a holistic standpoint. For instance, it could be that our pipelined approach (or underlying “annotator”) is proficient in detecting LIFE events, while underperforming on JUSTICE events.

### 5.3 Ethical considerations

The automated, synthetic annotation of datasets via LLMs entails several ethical considerations:

First, as LLMs increasingly outperform human annotators, any large-scale automated annotation of datasets may further reduce the demand for human annotation specialists, thus promoting the devaluation of expert manual labor and possibly bringing about socio-economic consequences.

Furthermore, although the LLM-generated annotations can be employed to promote the training (or distillation) of specialized expert models whose hardware, energy, and compute requirements (and therefore carbon footprint) are several orders of magnitude lower than those of the annotating models, the annotation procedure *itself* can introduce a considerable computational (and financial) overhead, depending on dataset volume, extent of annotation guidelines, model choice, and simulated annotators *A*_1_, ..., *A*_*k*_.

Moreover, the responses generated by LLMs can reproduce or exaggerate the (often subtle) biases inherent in their training data (Guo et al., 2024; Gallegos et al., 2024), potentially corrupting the model's annotations—especially regarding event arguments that involve (certain) people, places, or topics—as well as models trained on thus obtained datasets. To mitigate such cases, rigorous (and possibly manual) dataset auditing and proper rectification procedures may be appropriate.

## 6 Conclusion

This work examines the extent to which extensive, detailed annotation guidelines can improve an LLM's performance on event extraction. By introducing dataset-specific task instructions into a guideline-driven LLM prompting framework, we showcase that LLMs can greatly benefit from detailed guidelines when compared with commonly used bare-minimum instructions, assuming that datasets are guideline-compliant. Using the extensive ACE 2005 English Annotation Guidelines for Events as a reference document, performance on event detection for the ACE 2005 dataset can be improved by up to 6.7 percent F1 under the reasoning model o3-mini. While exact argument extraction remains a challenge, we demonstrate through soft-matching that, instead of determining an argument's *existence* and (approximate) textual position, the problem that remains is *accurate* boundary detection. This, we argue, might be fixable with additional guidelines on detecting said boundaries.

Furthermore, our experiments demonstrate that labeling datasets with LLMs can provide notable baselines (for training) when no human-labeled datasets are available, particularly when approximate argument boundaries are permissible. The experiments nevertheless underscore that LLM-generated annotations should not be viewed as a drop-in replacement for expert annotations.

Overall, our research provides valuable insights on labeling datasets with LLMs, and emphasizes detailed instructions as a promising yet underexplored element for in-context learning and LLM-based annotations. By directing attention toward more instructional and extensive task descriptions, our findings suggest a practical approach for leveraging LLMs as explainable, and guideline-compliant annotators.

## Data Availability

Publicly available datasets were analyzed in this study. This data can be found here: https://catalog.ldc.upenn.edu/LDC2006T06 (ACE 2005), https://github.com/ej0cl6/TextEE/ (MEE), and https://github.com/THU-KEG/MAVEN-dataset (MAVEN).

## Footnotes

1
https://www.ldc.upenn.edu/sites/www.ldc.upenn.edu/files/english-events-guidelines-v5.4.3.pdf

2
https://github.com/dwadden/dygiepp

3
https://blender.cs.illinois.edu/software/oneie/

4
http://nlp.uoregon.edu/download/MEE/

5 Surely, 1, 135 − 979 = 156, but one instance includes unresolvable argument boundaries.

6
https://github.com/THU-KEG/MAVEN-dataset

7
https://huggingface.co/meta-llama/Llama-3.3-70B-Instruct

8
https://openai.com/index/openai-o3-mini/

9
https://platform.openai.com/docs/guides/structured-outputs

10
https://github.com/noamgat/lm-format-enforcer

11
https://github.com/ej0cl6/TextEE

12
https://www.ldc.upenn.edu/collaborations/past-projects/ace/annotation-tasks-and-specifications
