# Supplementary material for: Improving LLM-based event extraction with annotation guidelines
Source: Front Artif Intell. 2026 Aug 13;9:1797435. doi: 10.3389/frai.2026.1797435 (PMC13518317; doi:10.3389/frai.2026.1797435)
Supplement: Supplementary file 1 [file Data_Sheet_1.pdf]

Supplementary Material

1 SUMMARIZED GUIDELINES: EVENT DETECTION

The complete $SAGE_{ED}$ document, summarizing the Event Detection-centered information formulated in the *ACE English Annotation Guidelines for Events* (AGE). The provided section numbers, e.g., §2 and §5, correspond to the original AGE document. The highlighted paragraphs (in grey) represent the examples that are incorporated into $SAGE_{ED}$ via the +E modifier described in our Experimental Settings.

1.1 Basic Concepts (§1)

Event: An occurrence or "something happening" involving participants.

Event Scope: The sentence that describes the Event.

Event Trigger: The word or phrase that best represents or expresses the Event. The Event Trigger determines the Event Scope.

Event Argument: An Entity or Attribute involved in the Event. An Event Argument must occur within the corresponding Event Scope.

Example: "[Alice] was *born* in [Berlin]. [She] used to *work* in [Chicago]." Here, 'born' and 'work' are annotated as Event Triggers. The word 'born' represents the Birth-Event (with 'Alice' and 'Berlin' as Event Arguments), and 'work' represents the Work-Event (with 'She' and 'Chicago' as Event Arguments).

1.2 Event Nominalizations and Pronominalizations (§2)

Single Role Rule: If a noun represents an Event participant (an Entity), such as 'nominee' or 'attacker', and simultaneously meets the requirements for indicating the occurrence of an Event, it should **not** be annotated as an Event Trigger.

Example: "The nominee is absent." Here, 'nominee' is an Event participant and should, therefore, not be annotated as an Event Trigger for the underlying NOMINATE-Event.

Missing Head Rule: If an adjective (or participle) in a modifier position is without an associated head noun, that word should **not** be annotated as an Event Trigger, because it will instead be treated as the missing head noun.

Example: "The injured were rushed to St John Macomb Medical Center." Here, 'injured' should not be annotated as an Event Trigger, because it assumes the function of the missing head noun (presumably 'people').

Verb Modifier Rule: If an Event is best represented by an adjective or participle that modifies the main verb (not a missing head noun), the adjective or participle may be annotated as an Event Trigger.

Example: "The crash left 20 *dead*." Here, 'dead' is an adjective modifying the main verb 'left' and can therefore be annotated as an Event Trigger.

1.3 Multiple Possible Triggers (§2)

Some Events may be represented by multiple words, complicating the identification of the Event Trigger. To address this issue, we introduce the following rules:

Stand-Alone Noun Rule: Some Events are expressed by combining the main verb with a noun. If the noun can represent the Event by *itself*, without requiring the verb, it should be annotated as the Event Trigger. Note: Mind the Single Role Rule.

Example: "Jane had to pay a *fine* of $200." Here, the main verb is 'pay', but the word that represents the FINE-Event (by itself) is 'fine', therefore it is annotated as the Event Trigger.

Stand-Alone Adjective Rule: Some Events are expressed by combining a main (support) verb with an adjective or participle that describes the resulting situation. If the adjective or participle can represent the Event by *itself*, without requiring the verb, it should be annotated as the Event Trigger. Note: Mind the Verb Modifier Rule.

Example: "The explosion left dozens *injured*." Here, the main (support) verb is 'left', but the word that describes the resulting situation (by itself) is 'injured', therefore it is annotated as the Event Trigger.

Verb Chain Rule: Some Events are expressed by chaining together multiple verbs. In these cases, the main verb (usually the last verb in the chain) should be annotated as the Event Trigger. The non-main verbs are usually support verbs, such as modal verbs (e.g., would and should), aspectual verbs (e.g., have and will), as well as the verb 'to be'.

Example: "John could be *sentenced* to 5 years." Here, the chain of verbs 'could be sentenced' represents a SENTENCE-Event, but only the main verb 'sentenced' is annotated as the Event Trigger.

Verb Particle Rule: Some Events are expressed by combining the main verb with a particle. If the main verb and particle are syntactically contiguous, they should be annotated as one multi-word Event Trigger. If, however, the main verb and particle are non-contiguous, only the main verb should be annotated as the Event Trigger.

Example 1: "Jane was *laid off* by XYZ." Here, the main verb 'laid' and the particle 'off' are contiguous and are therefore annotated as one multi-word Event Trigger.
Example 2: "XYZ *laid* Jane off." Here, the main verb 'laid' and the particle 'off' are non-contiguous, thus only the main verb is annotated as the Event Trigger.

1.4 Multiple Distinct Events (§2)

The presence of multiple potential triggers for the *same* Event must not be confused with the existence of multiple *distinct* Events.

Example: "Kennedy was *shot dead* by Oswald." Here, 'shot' and 'dead' represent two separate Events (ATTACK and DIE) and are, therefore, two individual Event Triggers.

In some cases, multiple Events are triggered by multiple resultative adjectives (or participles) that share the same support verb.

Example: "The explosion left at least 30 *dead* and dozens *injured*." Here, the adjectives 'dead' and 'injured' share the same support verb 'left', while representing separate Events.

Sometimes, the decision between singular or multiple Events is resolved by applying one of the Stand-Alone Rules.

Example 1: "An officer witnessed the *attack*." Here, the noun 'attack' can represent the Event by itself, hence it is the (only) Event Trigger. (Stand-Alone Noun Rule)
Example 2: "The explosion left 30 *dead*." Here, the adjective 'dead' can represent the Event by itself, hence it is the (only) Event Trigger. (Stand-Alone Adjective Rule)

If the Stand-Alone Rules are unable to resolve the decision by themselves, tests can be performed:

a) If the two Events are not caused by the same Entity, then two separate Events should be annotated.
b) If Event one is 'included' in Event two, then two separate Events should be annotated.
c) If Event one describes the 'internal structure' of Event two, then two separate Events should be annotated.
d) In cases of doubt, two separate Events should be annotated.

1.5 Polarity, Tense, Genericity, Modality (§3)

Polarity: Events are either NEGATIVE or POSITIVE. NEGATIVE Events are explicitly communicated as non-occurrences, typically indicated by negations or negative contexts. All other Events are POSITIVE.

Example 1: "John is unable to *visit* Jane." (NEGATIVE)
Example 2: "John will be *visiting* Jane." (POSITIVE)

Tense: Events can be expressed in PAST, FUTURE, or PRESENT tense. If an Event's tense cannot be determined through context, then it is UNSPECIFIED.

Example 1: "John plans to *visit* Jane." (FUTURE)
Example 2: "John's *visits* with Jane." (UNSPECIFIED)

Genericity: Events are either SPECIFIC or GENERIC. SPECIFIC Events are expressed as individual occurrences at specified times and places. All other Events are GENERIC.

Example 1: "In 1987, John *visited* Jane." (SPECIFIC)
Example 2: "John used to *visit* Jane." (GENERIC)

Modality: Events are either ASSERTED or OTHER. ASSERTED Events are expressed as factual occurrences. OTHER Events are expressed as believed, hypothetical, commanded, requested, threatened, proposed, discussed, desired, promised, or otherwise ambiguous occurrences.

Example 1: "Last year, John *visited* Jane." (ASSERTED)
Example 2: "John considers *visiting* Jane." (OTHER)

Important: An Event should be annotated **regardless** of its polarity (POSITIVE or NEGATIVE), tense (PAST, PRESENT, or FUTURE), genericity (SPECIFIC or GENERIC), or modality (ASSERTED or OTHER).

Example: "John will not *visit* Jane." Here, 'visit' is an Event Trigger, despite the future tense and negation (i.e., negative polarity).

1.6 Taggable Events (§5)

Only the following Events should be annotated: LIFE, MOVEMENT, TRANSACTION, BUSINESS, CONFLICT, CONTACT, PERSONNEL, and JUSTICE. All unrelated Events should be ignored.

Events are only 'taggable' if one of their respective scenarios is satisfied.

1.6.1 Event Type: LIFE

- *BE-BORN* – A person is given birth to. (triggered by *born*, *birth*, etc.)
- *MARRY* – Two people are legally married. (triggered by *married*, *marriage*, etc.)
- *DIVORCE* – Two people are legally divorced. (triggered by *divorce*, *divorced*, etc.)
- *INJURE* – A person is physically harmed. (triggered by *wounded*, *injured*, *hurt*, *hospitalized*, *disabled*, etc.)
- *DIE* – A person's life ends. (triggered by *assassinate*, *assassination*, *kill*, *killed*, *slain*, *died*, *dead*, *death*, *fatal*, *suicide*, etc.)

1.6.2 Event Type: MOVEMENT

- *TRANSPORT* – The movement of people or artifacts. (triggered by *transport*, *moved*, *remove*, *travel*, etc.)

1.6.3 Event Type: TRANSACTION

- *TRANSFER-OWNERSHIP* – The ownership of artifacts or organizations is transferred via borrowing, donations, or transactions. (triggered by *purchase*, *acquire*, *got*, *sell*, *selling*, *buy*, *buying*, etc.)
- *TRANSFER-MONEY* – A financial transaction not associated with purchasing something. (triggered by *receive*, *giving*, *loaning*, *donated*, *donations*, *borrowed*, etc.)

1.6.4 Event Type: BUSINESS

- *START-ORG* – A new organization is created or founded. (triggered by *founded*, *launched*, *organize*, etc.)
- *MERGE-ORG* – Two or more organizations are merged or combined. (triggered by *merger*, *merged*, etc.)
- *DECLARE-BANKRUPTCY* – An entity declares bankruptcy. (triggered by *Chapter 11*, *bankrupt*, *bankruptcy*, etc.)
- *END-ORG* – An organization is permanently closed or dissolved. (triggered by *folded*, etc.)

1.6.5 Event Type: CONFLICT

- *ATTACK* – A violent physical act possibly causing harm or damage. (triggered by *bomb*, *exploded*, *gunfire*, *clashes*, *coup*, *attacks*, *blew*, *firing*, *battle*, *threw*, etc.)
- *DEMONSTRATE* – A public gathering of people demonstrating. (triggered by *rioted*, *rioting*, *strike*, *rallied*, *demonstrated*, *protesting*, *took to the town's streets*, *protests*, *walkout*, *stoppage*, etc.)

1.6.6 Event Type: CONTACT

- *MEET* – An in-person (face-to-face) meeting between entities, including gatherings, talks, visits, summits, and conferences. (triggered by *meet*, *met*, *meeting*, *summit*, etc.)
- *PHONE-WRITE* – A discussion via written or telephone communication (not face-to-face) between at least two specified entities. (triggered by *sent*, *discussed*, *teleconference*, *called*, *dash*, *communicate*, *interact*, etc.)

1.6.7 Event Type: PERSONNEL

- *START-POSITION* – A person begins working for an organization or geopolitical entity by employment, changing positions, election, or otherwise. (triggered by *joined*, *hire*, *hired*, *started office*, *succeeded*, etc.)
- *END-POSITION* – A person stops working for an organization or geopolitical entity by leaving, retiring, changing positions, or otherwise. (triggered by *succeeded*, *fired*, *laid off*, etc.)
- *NOMINATE* – A person is officially proposed or nominated for employment in an organization or geopolitical entity. (triggered by *nominated*, *nomination*, etc.)
- *ELECT* – A person is elected to an office or position in an organization or geopolitical entity. (triggered by *elected*, *election*, etc.)

1.6.8 Event Type: JUSTICE

- *ARREST-JAIL* – A person is officially detained (in custody) or incarcerated by an organization or state actor. (triggered by *jailed*, *incarcerated*, *imprisoned*, *arrested*, etc.)
- *RELEASE-PAROLE* – A person is officially released from custody because the sentence has been completed, charges have been dismissed, or parole has been granted. (triggered by *released*, *freed*, etc.)
- *TRIAL-HEARING* – A court proceeding is initiated to determine an entity's guilt or innocence in a criminal offense. OR: A formal gathering (e.g., hearings) of state actors to discuss a criminal legal situation. (triggered by *tried*, *trial*, *hearing*, etc.)

- *CHARGE-INDICT* – An entity is formally accused of committing a criminal offense by a state actor. OR: A state actor engages in official legal action to prosecute an allegation. (triggered by *indicted*, *indictment*, *charged*, etc.)
- *SUE* – A court proceeding is initiated to resolve a dispute or determine an entity's liability through legal claims or lawsuits. (triggered by *suit*, *sue*, *lawsuit*, etc.)
- *CONVICT* – An entity is convicted (found guilty) or pleaded guilty. (triggered by *convicted*, *guilty*, etc.)
- *SENTENCE* – An entity is formally punished by a state actor (e.g., incarcerated). (triggered by *sentenced*, etc.)
- *FINE* – An entity is fined by a state actor. (triggered by *fine*, *fined*, etc.)
- *EXECUTE* – An entity is formally executed by a state actor. (triggered by *executed*, *execution*, etc.)
- *EXTRADITE* – A person is extradited by a state actor. (triggered by *extradited*, *extradition*, etc.)
- *ACQUIT* – A legal proceeding is concluded without a conviction. (triggered by *acquitted*, etc.)
- *APPEAL* – A court decision is appealed. (triggered by *appeal*, etc.)
- *PARDON* – A sentence is pardoned by a head-of-state or representative. (triggered by *pardon*, etc.)

2 SUMMARIZED GUIDELINES: ARGUMENT EXTRACTION

The complete SAGE_{EAE} document, summarizing the Argument Extraction-centered information formulated in the *ACE English Annotation Guidelines for Events* (AGE). The provided section numbers, e.g., §6.1, correspond to the original AGE document. The highlighted paragraphs (in grey) represent the examples that are incorporated into SAGE_{EAE} via the +E modifier described in Experimental Settings.

2.1 Basic Concepts (§1)

Event: An occurrence or "something happening" involving participants.

Event Scope: The sentence that describes the Event.

Event Trigger: The word or phrase that best represents or expresses the Event. The Event Trigger determines the Event Scope.

Event Argument: An Entity or Attribute involved in the Event. An Event Argument must occur within the corresponding Event Scope.

Example: "[Alice] was *born* in [Berlin]. [She] used to *work* in [Chicago]." Here, 'born' and 'work' are annotated as Event Triggers. The word 'born' represents the Birth-Event (with 'Alice' and 'Berlin' as Event Arguments), and 'work' represents the Work-Event (with 'She' and 'Chicago' as Event Arguments).

2.2 Basic Rules (§6.1)

The following describes the basic rules for annotating Event Arguments.

Argument Scope: An argument (i.e., Entity or Value) should only be annotated if it occurs within the Event Scope of the proposed Event. In addition, we select the arguments by closeness (syntactic proximity) to the Event Trigger.

Example: "Jane shouted as she watched [John] *steal* [her] painting." Here, 'her' (instead of 'Jane') is annotated as an argument (e.g., VICTIM-ARG) due to its syntactic proximity to 'steal'.

Modal Certainty: An argument (i.e., Entity or Value) should be annotated *even* if its involvement in the proposed Event is hypothetical, uncertain, or speculative.

Example: "[Einhorn] was accused of *killing* Maddux." Here, 'Einhorn' should be annotated as an argument (e.g., KILLER-ARG) for the Event indicated by 'killing', despite the uncertainty introduced by the word 'accused'.

Reasonable Reader: An Entity or Value should only be annotated as an argument for the proposed Event if it *cannot* reasonably be interpreted as uninvolved, given the respective modality context.

Example: "Alice told Bob and Eve about the *stolen* painting." Here, neither Alice, Bob, nor Eve can be annotated as an argument (e.g., THIEF-ARG) for the Event indicated by 'stolen', because none of them can reasonably be considered to be involved in the Event (irrespective of modality context, see **Modal Certainty**).

Shared Arguments: If an Entity or Value is involved in multiple Events, it should be annotated as an argument for each of them.

Example: "The *explosion* in [Lahore] *wounded* 36 people." Here, 'Lahore' should be annotated as an argument (e.g., PLACE-ARG) for the Events indicated by 'explosion' and 'wounded', respectively.

Related Event Mentions: When an Event is mentioned *alongside* its taggable Sub-Events, all possible valid arguments of the "Main" Event should be annotated, *unless* those arguments are understood to apply more directly to one of the Sub-Events.

Example: "Six *murders* took place in [France], including the *killing* of [Bob] and the *homicide* of [Joe]." Here, 'France' should be annotated for each of the proposed Events, while 'Bob' and 'Joe' are specific to the Sub-Events indicated by 'killing' and 'homicide', respectively.

PLACE-ARG Arguments: An Entity may be annotated as the PLACE-ARG argument *even* if the proposed Event takes place *at* or *near* that Entity.

Example: "A car bomb *went off* outside [army headquarters]." Here, 'army headquarters' is annotated as an argument (PLACE-ARG), even though the Event takes place 'outside' that location.

TIME-ARG Arguments: Only TIMEX2 mentions that occur within the relevant Event Scope and fulfill the **Reasonable Reader** clause should be annotated (or *timestamped*) as TIME-ARG arguments. If several Events can be equally associated with some TIMEX2 mention, that TIMESTAMP should only be

associated with the closest Event (by measure of syntactic proximity), *unless* contextual evidence suggests otherwise.

> **Example:** "Having *missed* the [Friday] deadline, Sarah proposed a *meeting*." Here, due to its syntactic proximity, 'Friday' should be annotated as the timestamp (i.e., TIME-ARG) for the Event indicated by 'missed', not the proposed 'meeting'.
